# Metabolomics reveals a link between homocysteine and lipid metabolism and leukocyte telomere length: the ENGAGE consortium

**DOI:** 10.1038/s41598-019-47282-6

**Published:** 2019-08-12

**Authors:** Ashley van der Spek, Linda Broer, Harmen H. M. Draisma, René Pool, Eva Albrecht, Marian Beekman, Massimo Mangino, Mait Raag, Dale R. Nyholt, Harish K. Dharuri, Veryan Codd, Najaf Amin, Eco J. C. de Geus, Joris Deelen, Ayse Demirkan, Idil Yet, Krista Fischer, Toomas Haller, Anjali K. Henders, Aaron Isaacs, Sarah E. Medland, Grant W. Montgomery, Simon P. Mooijaart, Konstantin Strauch, H. Eka D. Suchiman, Anika A. M. Vaarhorst, Diana van Heemst, Rui Wang-Sattler, John B. Whitfield, Gonneke Willemsen, Margaret J. Wright, Nicholas G. Martin, Nilesh J. Samani, Andres Metspalu, P. Eline Slagboom, Tim D. Spector, Dorret I. Boomsma, Cornelia M. van Duijn, Christian Gieger

**Affiliations:** 1 Department of Epidemiology, Erasmus Medical Center, Rotterdam, The Netherlands; 2 Department of Internal Medicine, Erasmus Medical Center, Rotterdam, The Netherlands; 3 Department of Biological Psychology, VU University Amsterdam, Amsterdam, The Netherlands; 4 Amsterdam Public Health research institute, Amsterdam University Medical Centers, Amsterdam, The Netherlands; 5 Section of Genomics of Common Disease, Imperial College London, Burlington Danes Building Room E301, Du Cane Road, London, W12 0NN UK; 6 BBMRI-NL: Infrastructure for the Application of Metabolomics Technology in Epidemiology (RP4), Utrecht, The Netherlands; 7 Institute of Genetic Epidemiology, Helmholtz Zentrum München - German Research Center for Environmental Health, Neuherberg, Germany; 8 Molecular Epidemiology, Department of Biomedical Data Sciences, Leiden University Medical Center, Leiden, The Netherlands; 9 Department of Twin Research and Genetic Epidemiology, King’s College London, London, UK; 10 NIHR Biomedical Research Centre at Guy’s and St Thomas’ Foundation Trust, London, SE1 9RT UK; 11 Institute of Family Medicine and Public Health, University of Tartu, Tartu, Estonia; 12 School of Biomedical Sciences, Faculty of Health, Institute of Health and Biomedical Innovation, Queensland University of Technology, Brisbane, QLD Australia; 13 Department of Human Genetics, Leiden University Medical Center, Leiden, The Netherlands; 14 Department of Cardiovascular Sciences, University of Leicester and NIHR Leicester Biomedical Research Centre, Glenfield Hospital, Leicester, UK; 15 Max Planck Institute for Biology of Ageing, Cologne, Germany; 16 Department of Bioinformatics, Institute of Health Sciences, Hacettepe University, 06100, Ankara, Turkey; 17 Estonian Genome Center, Institute of Genomics, University of Tartu, Tartu, Estonia; 18 Institute of Mathematics and Statistics, University of Tartu, Tartu, Estonia; 19 The Institute for Molecular Biosciences, The University of Queensland, Brisbane, Australia; 20 CARIM School for Cardiovascular Diseases, Maastricht Centre for Systems Biology (MaCSBio), and Department of Biochemistry, Maastricht University, Maastricht, The Netherlands; 21 QIMR Berghofer Medical Research Institute, Brisbane, Australia; 22 Department of Gerontology and Geriatrics, Leiden University Medical Center, Leiden, The Netherlands; 23 Chair of Genetic Epidemiology, IBE, Faculty of Medicine, LMU Munich, Germany; 24 Research Unit of Molecular Epidemiology, Helmholtz Zentrum München - German Research Center for Environmental Health, Neuherberg, Germany; 25 Queensland Brain Institute, The University of Queensland, Brisbane, Australia; 26 Leiden Academic Centre for Drug Research, Leiden University, Leiden, Netherlands; 27 Nuffield Department of Population Health, University of Oxford, Oxford, UK

**Keywords:** Metabolomics, Telomeres

## Abstract

Telomere shortening has been associated with multiple age-related diseases such as cardiovascular disease, diabetes, and dementia. However, the biological mechanisms responsible for these associations remain largely unknown. In order to gain insight into the metabolic processes driving the association of leukocyte telomere length (LTL) with age-related diseases, we investigated the association between LTL and serum metabolite levels in 7,853 individuals from seven independent cohorts. LTL was determined by quantitative polymerase chain reaction and the levels of 131 serum metabolites were measured with mass spectrometry in biological samples from the same blood draw. With partial correlation analysis, we identified six metabolites that were significantly associated with LTL after adjustment for multiple testing: lysophosphatidylcholine acyl C17:0 (lysoPC a C17:0, *p-value* = 7.1 × 10^−6^), methionine (*p-value* = 9.2 × 10^−5^), tyrosine (*p-value* = 2.1 × 10^−4^), phosphatidylcholine diacyl C32:1 (PC aa C32:1, *p-value* = 2.4 × 10^−4^), hydroxypropionylcarnitine (C3-OH, *p-value* = 2.6 × 10^−4^), and phosphatidylcholine acyl-alkyl C38:4 (PC ae C38:4, *p-value* = 9.0 × 10^−4^). Pathway analysis showed that the three phosphatidylcholines and methionine are involved in homocysteine metabolism and we found supporting evidence for an association of lipid metabolism with LTL. In conclusion, we found longer LTL associated with higher levels of lysoPC a C17:0 and PC ae C38:4, and with lower levels of methionine, tyrosine, PC aa C32:1, and C3-OH. These metabolites have been implicated in inflammation, oxidative stress, homocysteine metabolism, and in cardiovascular disease and diabetes, two major drivers of morbidity and mortality.

## Introduction

Telomeres are located at the ends of chromosomes and protect against spontaneous DNA damage, thus preserving genomic integrity^1,2^. The progressive shortening of telomere length with each subsequent cell division underlies the so-called mitotic clock, i.e. the limited replicative capacity of a cell^3^. Replicative senescence and subsequent cell death occurs when the mean telomere length reaches a critical value and telomere length is therefore seen as a marker for biological age^4–6^. Short leukocyte telomere length (LTL) has been associated with age^5,7–9^ and multiple age-related diseases such as cardiovascular disease^10–15^, diabetes^10,16,17^ and dementia^18,19^. Short LTL has also been associated with mortality^20–27^, although not all studies support this association^28–33^. However, the biological mechanisms underlying the associations of LTL with age-related diseases and mortality are still largely unknown.

Longevity in humans has previously been associated with various metabolic traits in the elderly, including traits related to blood pressure and lipids, suggesting that changes at the metabolic level are key features in longevity^34–36^. Metabolic profiles have been associated with age and various age-related diseases, such as type 2 diabetes, atherosclerosis, cancer, and Alzheimer’s Disease^37–44^. Only a few studies investigated the association of metabolic markers with LTL, all using untargeted metabolomics^45–47^. One study focused on metabolic markers involved in aging and early development in 6,055 individuals included in the TwinsUK registry^45^. Although a combined set of 22 metabolites was strongly correlated with age and age-related traits, the individual metabolites were not significantly associated with LTL^45^. Another study identified 19 metabolites associated with LTL in a small sample of American Indians (n = 423)^46^. The most recent study was conducted in 3,511 females from the TwinsUK registry, reporting associations of five metabolites with LTL. These include gamma-glutamyltyrosine, gamma-glutamylphenylalanine, 1-stearoylglycerophosphoinositol, 1-palmitoylglycerophosphoinositol, and 4-vinylphenol sulfate^47^.

In the current study, we used a standardized targeted metabolomics approach to investigate the association between LTL and serum metabolites of key biochemical pathways in the largest sample so far consisting of 7,853 individuals from seven independent population-based cohorts from Europe and Australia. We further aimed to disentangle the metabolic pathways that are represented by the metabolites significantly associated with LTL.

## Methods

### Cohort descriptions and measurements of LTL and metabolites

The cohorts included in this study are the Cooperative Health Research in the Region of Augsburg (KORA) study, the Netherlands Twin Register (NTR), the Estonian Genome Center University of Tartu (EGCUT) study, the TwinsUK cohort, the Erasmus Rucphen Family (ERF) study, the Leiden Longevity Study (LLS), and the Queensland Institute of Medical Research (QIMR) study, all part of the ENGAGE consortium. Details on the individual cohorts as well as details on the LTL quantitative polymerase chain reaction measurements and the metabolites as measured with the Absolute*IDQ*^TM^ p150 kit (BIOCRATES Life Sciences AG, Innsbruck, Austria) are provided in the Supplementary Materials. In summary, both LTL and metabolite concentrations were measured in the same laboratories according to a common protocol, using blood samples taken at the same time point. To ensure good data quality, each metabolite had to meet three criteria for inclusion in the study: 1) coefficient of variation (CV) not exceeding 25%; 2) less than 5% missing values; 3) median of metabolite concentrations above the limit of detection. This quality control was performed per cohort. Supplementary Table 1 describes the reasons for exclusion of metabolites from the analysis for each cohort. The metabolites measured include hexoses (H1), amino acids (AA), acyl-carnitines (AC), sphingomyelins (SMs), diacyl phosphatidylcholines (PC aa), acyl-alkyl-phosphatidylcholines (PC ae) and lysophosphatidylcholines (lysoPC).

Written informed consent was obtained from all study participants. The study protocol was approved by the medical ethics boards of the Helmholtz Zentrum München, VUmc Amsterdam, University of Tartu, St. Thomas’ Hospital London, Erasmus MC Rotterdam, LUMC Leiden, and Queensland Institute of Medical Research and all investigations were carried out in accordance with the Declaration of Helsinki.

### Statistical analysis

To standardize LTL measurements across cohorts we Z-transformed the LTL values. Metabolite concentration values were natural log-transformed to attain a better approximation of the normal distribution. We performed partial correlation analysis per cohort, adjusting for age and sex, and if necessary for family relationships (model 1). In the extended model (model 2) we additionally adjusted for body mass index (BMI). We performed a sensitivity analysis to test the robustness of the results and repeated the meta-analysis excluding studies with low sample size or high mean LTL values.

A multiple testing-corrected statistical significance threshold for association of metabolite concentrations with LTL was defined at the meta-analysis level. We accounted for multiple testing by Bonferroni correction based on the effective number of independent variables (VeffLi) in the metabolite concentration data^48^ (https://neurogenetics.qimrberghofer.edu.au/matSpD/). The VeffLi value was determined using the correlation matrix of the quality controlled and log-transformed metabolomics data in the ERF and NTR cohorts, yielding a VeffLi (representing the number of independent metabolites) of 46 for both cohorts. This resulted in a Bonferroni corrected significance threshold of *p-value* < 1.1 × 10^−3^ (=0.05/46).

### Pathway analysis of the associated metabolites

For interpretation of the observed associations we followed two bioinformatics approaches in parallel. First, we employed a bioinformatics pipeline based on a workflow management software tool called “Taverna” (http://www.taverna.org.uk)^49^ to determine if the metabolites associated with LTL share a network space within two reaction steps. We took as input all possible pairs of significantly associated metabolites. In this pipeline, all the reactions within a radius of two steps in the reaction space of the first metabolite were obtained from the KEGG database^50^. The second metabolite is searched against the substrates and the products of the reactions obtained in the previous step. The presence of the second metabolite in any of the reaction steps is an indication that the two metabolites participate in reactions within two steps of each other. In the final step, the path between the two metabolites is returned to the user. In order to prevent non-specific connections, an intermediate step filters out hub metabolites such as ATP, ADP, and NADP. Next, we used the function “heatmap.2” included in the R package “gplots” (https://CRAN.R-project.org/package=gplots) to prepare a heat map of the correlation of the individual metabolites with LTL in models 1 and 2. For this analysis, default functions for clustering were used (distance measure: euclidean distance). We also derived a correlation matrix for the significantly associated metabolites in ERF and visualized this in a correlogram using the R package “corrplot”^51^.

## Results

General characteristics of the study populations are provided in Table 1. The study covers a wide age range, with the mean age of the participants ranging from 18.4 to 62.9 years. Most studies had approximately equal numbers of males and females, except for NTR (33% female) and TwinsUK (only females). Mean LTL ranged from 1.44 (LLS) to 3.58 (TwinsUK). BMI was on average between 25.2 and 27.6 kg/m^2^, but was unavailable at the time of metabolite and LTL assessment in the QIMR study.

**Table 1:** General characteristics of the study populations.

	n	n_BMI	% female	LTL			Age			BMI		
				mean	SD	95% CI	mean	SD	95% CI	mean	SD	95% CI
KORA	3003	2988	51.8	1.85	0.33	1.84 - 1.86	56.08	13.25	55.61 - 56.55	27.61	4.80	27.44 - 27.78
NTR	1314	1307	33.3	2.54	0.47	2.51 - 2.57	50.60	14.13	49.84 - 51.36	25.97	3.80	25.76 - 26.18
EGCUT	1084	1084	50.3	1.90	0.30	1.88 - 1.92	37.78	15.70	36.85 - 38.71	25.16	4.56	24.89 - 25.43
TwinsUK	810	810	100.0	3.58	0.64	3.54 - 3.62	53.72	10.76	53.10 - 54.34	26.44	5.35	26.13 - 26.75
ERF	806	806	53.7	1.79	0.37	1.76 - 1.82	47.76	13.97	46.80 - 48.72	27.17	4.81	26.84 - 27.50
LLS	643	643	50.1	1.44	0.27	1.42 - 1.46	62.91	6.61	62.40 - 63.42	26.65	4.01	26.34 - 26.96
QIMR	193	0	48.2	3.43	0.56	3.35 - 3.51	18.44	12.65	16.66 - 20.22	NA	NA	NA

Abbreviations: n = number of participants with data available on metabolites, telomere length, age, and sex; n_BMI = number of participants with data available on metabolites, telomere length, age, sex, and BMI; LTL = leukocyte telomere length; SD = standard deviation. LTL as a ratio of telomere repeat length to copy number of the single copy gene *36B4*; Age in years; BMI in kg/m^2^.

Out of the 131 metabolites that passed quality control, 27 showed at least nominally significant correlation (*p-value < *0.05) with LTL when adjusting for age and sex in model 1 (Table 2). Six metabolites surpassed the multiple-testing corrected significance threshold (*p-value* < 1.1 × 10^−3^). Five of these metabolites were consistently associated with LTL in the same direction in at least five out of seven studies: lysophosphatidylcholine acyl C17:0 (lysoPC a C17:0, r = 0.05, *p-value* = 7.1 × 10^−6^) and phosphatidylcholine acyl-alkyl C38:4 (PC ae C38:4, r = 0.04, *p-value* = 9.0 × 10^−4^) were positively associated with LTL, while methionine (Met, r = −0.04, *p-value* = 9.2 × 10^−5^), tyrosine (Tyr, r = −0.04, *p-value* = 2.1 × 10^−4^), and phosphatidylcholine diacyl C32:1 (PC aa C32:1, r = −0.04, *p-value* = 2.4 × 10^−4^) were negatively associated with LTL. Although hydroxypropionylcarnitine (C3-OH, r = −0.10, *p-value* = 2.6 × 10^−4^) was also found negatively associated with LTL, this effect was only based on two out of seven studies. Additionally adjusting for BMI in model 2 had limited effect on the correlation coefficients of the six significant metabolites in model 1 and all metabolites except PC ae C38:4 remained significantly associated with LTL (Table 2). The summary statistics for all metabolite-LTL correlations for both models can be found in Supplementary Table 2. Study-specific results for the age- and sex-adjusted model are provided in Supplementary Table 3.

**Table 2:** Partial correlation meta-analysis results of LTL and metabolites (*p-value* in model 1 < 0.05).

Metabolite	Model 1: age + sex					Model 2: age + sex + BMI					Metabolite full name
	n	direction*	r	p-value	FDR	n	direction*	r	p-value	FDR	
lysoPC a C17:0	7853	++−++++	0.05	**7.1 × 10** ^**−6**^	9.3 × 10^−4^	7638	++−+++	0.04	**4.7 × 10** ^**−4**^	6.9 × 10^−3^	Lysophosphatidylcholine acyl C17:0
Met	7852	−−−−−−+	−0.04	**9.2 × 10** ^**−5**^	6.0 × 10^−3^	7637	−−−−−−	−0.05	**7.5 × 10** ^**−5**^	9.3 × 10^−4^	Methionine
Tyr	7047	−−−−?−+	−0.04	**2.1 × 10** ^**−4**^	6.9 × 10^−3^	6832	−−−−?−	−0.04	**8.9 × 10** ^**−4**^	6.9 × 10^−3^	Tyrosine
PC aa C32:1	7851	−−−−+−+	−0.04	**2.4 × 10** ^**−4**^	6.9 × 10^−3^	7636	−−−−++	−0.04	**3.4 × 10** ^**−4**^	6.9 × 10^−3^	Phosphatidylcholine diacyl C32:1
C3-OH	1449	????−−?	−0.10	**2.6 × 10** ^**−4**^	6.9 × 10^−3^	1449	????−−	−0.10	**2.7 × 10** ^**−4**^	6.0 × 10^−3^	Hydroxypropionylcarnitine
PC ae C38:4	7853	+−+−+++	0.04	**9.0 × 10** ^**−4**^	2.0 × 10^−2^	7638	+−+−++	0.03	4.7 × 10^−3^	3.1 × 10^−2^	Phosphatidylcholine acyl-alkyl C38:4
PC ae C40:3	7853	+++++++	0.04	1.6 × 10^−3^	3.0 × 10^−2^	7638	++++++	0.03	8.5 × 10^−3^	3.8 × 10^−2^	Phosphatidylcholine acyl-alkyl C40:3
PC ae C40:5	7853	+−+++++	0.04	1.9 × 10^−3^	3.1 × 10^−2^	7638	+−++++	0.03	1.9 × 10^−2^	1.0 × 10^−1^	Phosphatidylcholine acyl-alkyl C40:5
SM C20:2	7853	+−+−+++	0.03	2.4 × 10^−3^	3.5 × 10^−2^	7638	+−+−++	0.03	2.7 × 10^−3^	2.0 × 10^−2^	Sphingomyeline C20:2
C9	5262	+??−++ ?	0.04	2.9 × 10^−3^	3.8 × 10^−2^	5247	+??−++	0.04	1.1 × 10^−2^	7.5 × 10^−2^	Nonaylcarnitine
PC ae C40:4	7853	+0+++++	0.03	4.1 × 10^−3^	4.9 × 10^−2^	7638	+−++++	0.03	2.8 × 10^−2^	1.1 × 10^−1^	Phosphatidylcholine acyl-alkyl C40:4
PC aa C38:3	7852	−−−−+++	−0.03	7.0 × 10^−3^	7.5 × 10^−2^	7637	−−−−++	−0.02	3.5 × 10^−2^	1.3 × 10^−1^	Phosphatidylcholine diacyl C38:3
PC ae C36:1	7852	+−+−+++	0.03	7.4 × 10^−3^	7.5 × 10^−2^	7637	+−+−++	0.02	5.8 × 10^−2^	2.1 × 10^−1^	Phosphatidylcholine acyl-alkyl C36:1
PC aa C36:1	7850	−−−−+++	−0.03	9.3 × 10^−3^	8.3 × 10^−2^	7635	−−−−++	−0.03	4.0 × 10^−3^	3.0 × 10^−2^	Phosphatidylcholine diacyl C36:1
PC ae C40:6	7853	+−+−+++	0.03	9.5 × 10^−3^	8.3 × 10^−2^	7638	+−+−++	0.02	8.5 × 10^−2^	2.4 × 10^−1^	Phosphatidylcholine acyl-alkyl C40:6
SM (OH) C16:1	7047	+−+−?++	0.03	1.1 × 10^−2^	9.4 × 10^−2^	6832	+−+−?+	0.02	5.3 × 10^−2^	1.6 × 10^−1^	Hydroxysphingomyeline C16:1
C2	7853	−−+−−−+	−0.03	1.4 × 10^−2^	1.0 × 10^−1^	7638	−−+−−−	−0.03	1.5 × 10^−2^	8.3 × 10^−2^	Acetylcarnitine
PC ae C36:2	7853	++−−+++	0.03	1.4 × 10^−2^	1.0 × 10^−1^	7638	+−−−++	0.02	1.3 × 10^−1^	2.4 × 10^−1^	Phosphatidylcholine acyl-alkyl C36:2
PC ae C38:3	7852	+++−+++	0.03	1.7 × 10^−2^	1.1 × 10^−1^	7637	+++−++	0.02	3.6 × 10^−2^	1.3 × 10^−1^	Phosphatidylcholine acyl-alkyl C38:3
PC aa C42:0	7853	+++++++	0.03	2.0 × 10^−2^	1.3 × 10^−1^	7638	+++−++	0.02	8.9 × 10^−2^	2.4 × 10^−1^	Phosphatidylcholine diacyl C42:0
PC aa C32:0	7853	−−−−+++	−0.03	2.1 × 10^−2^	1.3 × 10^−1^	7638	−−−−++	−0.03	5.9 × 10^−3^	3.5 × 10^−2^	Phosphatidylcholine diacyl C32:0
PC aa C40:5	7849	−−−−+++	−0.03	2.2 × 10^−2^	1.3 × 10^−1^	7634	−−−−++	−0.03	1.7 × 10^−2^	8.3 × 10^−2^	Phosphatidylcholine diacyl C40:5
PC aa C38:1	836	?????++	0.08	2.6 × 10^−2^	1.5 × 10^−1^	643	?????+	0.05	1.7 × 10^−1^	2.4 × 10^−1^	Phosphatidylcholine diacyl C38:1
PC aa C36:2	7853	−−−−−++	−0.02	3.0 × 10^−2^	1.6 × 10^−1^	7638	−−−−−+	−0.03	1.0 × 10^−2^	4.9 × 10^−2^	Phosphatidylcholine diacyl C36:2
PC aa C34:1	7852	−−−−+++	−0.02	3.1 × 10^−2^	1.6 × 10^−1^	7637	−−−−++	−0.03	1.3 × 10^−2^	7.5 × 10^−2^	Phosphatidylcholine diacyl C34:1
PC ae C42:4	7853	+−+++++	0.02	3.5 × 10^−2^	1.8 × 10^−1^	7638	+−++++	0.01	2.1 × 10^−1^	2.5 × 10^−1^	Phosphatidylcholine acyl-alkyl C42:4
SM C26:0	5454	+??−+++	0.03	4.3 × 10^−2^	2.1 × 10^−1^	5246	+??−++	0.02	2.3 × 10^−1^	2.6 × 10^−1^	Sphingomyeline C26:0

Abbreviations: n = number of participants, r = correlation coefficient, BMI = body mass index, FDR = false discovery rate.

*Order of cohorts in direction column: KORA, NTR, EGCUT, TwinsUK, ERF, LLS, QIMR; Direction of effect represented by − (negative correlation) + (positive correlation) or ? (not included) for each study. Bold *p-values*: associations surpassing significance threshold (*p-value* < 1.1 × 10^-3^).

We next conducted a sensitivity analysis to determine whether the analyses were driven by a single cohort. We removed two cohorts from the analysis: the TwinsUK cohort, which had a high mean LTL ($\bar{x}{}_{{\rm{LTL}}}=3.58$) and the QIMR cohort, which had a small sample size (N = 193) and was on average younger than the other cohorts. After excluding the TwinsUK cohort from the meta-analysis, all metabolites remained significantly associated with LTL, except for PC aa C32:1 (*p-value* = 1.1 × 10^−3^) (Supplementary Table 4). All metabolites, except for PC ae C38:4 (*p-value* = 1.8 × 10^−3^), remained significantly associated with LTL after excluding the QIMR cohort from the meta-analysis (Supplementary Table 5).

**Figure 1:**
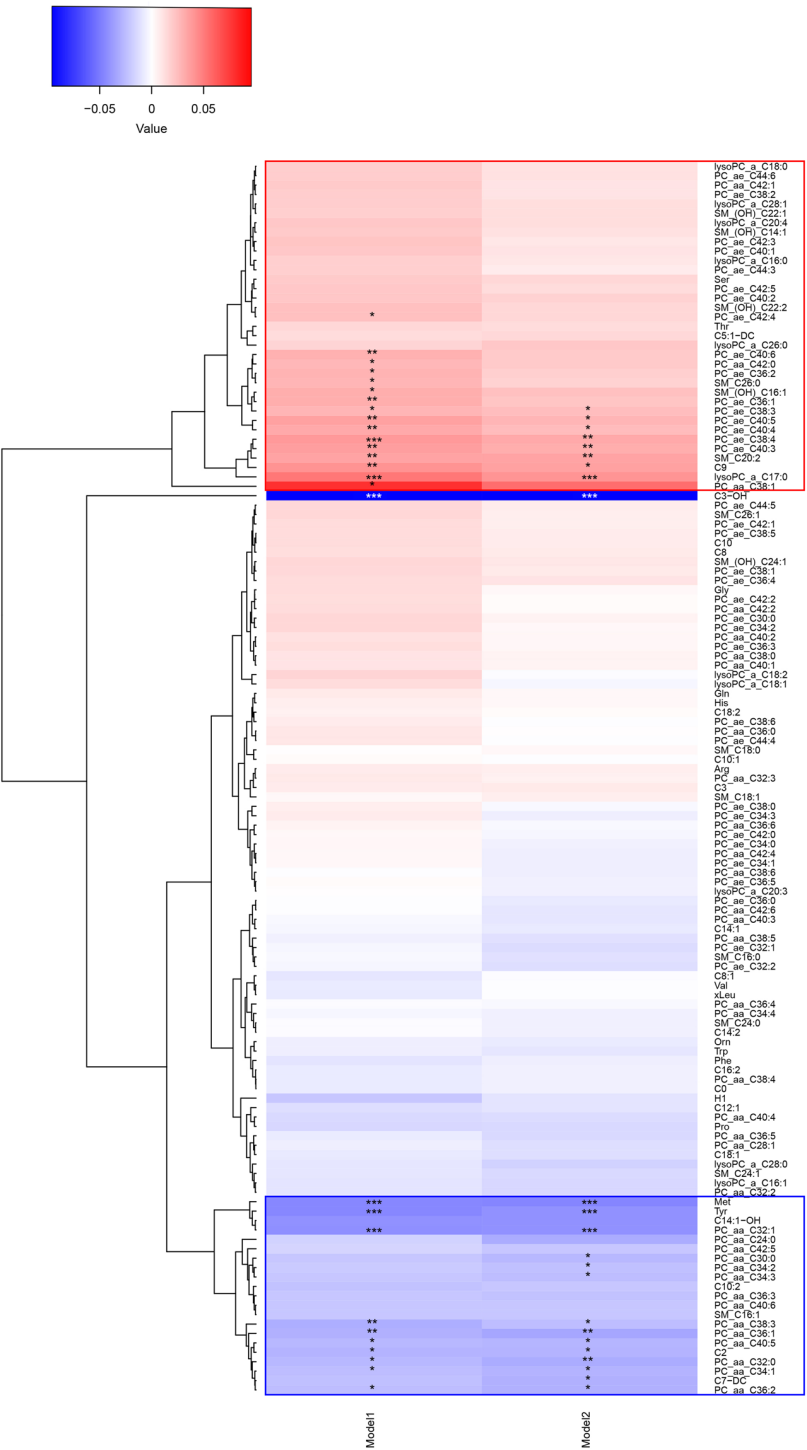
Heat map showing the results of the cluster analysis of metabolite correlations with LTL. The two statistical models used in our analysis are shown on the horizontal axis and all individual metabolites are depicted on the vertical axis. In model 1 we investigated the association of the metabolites with LTL adjusting for age and sex and in model 2 we additionally adjusted for BMI. A blue color indicates a negative partial correlation point estimate, while a red color indicates a positive partial correlation point estimate. Cluster 1 is shown by a red rectangle and cluster 2 by a blue rectangle. The stars represent the significance: *p-value < 0.05; **p-value < 0.01; ***p-value < 0.001.

**Figure 2:**
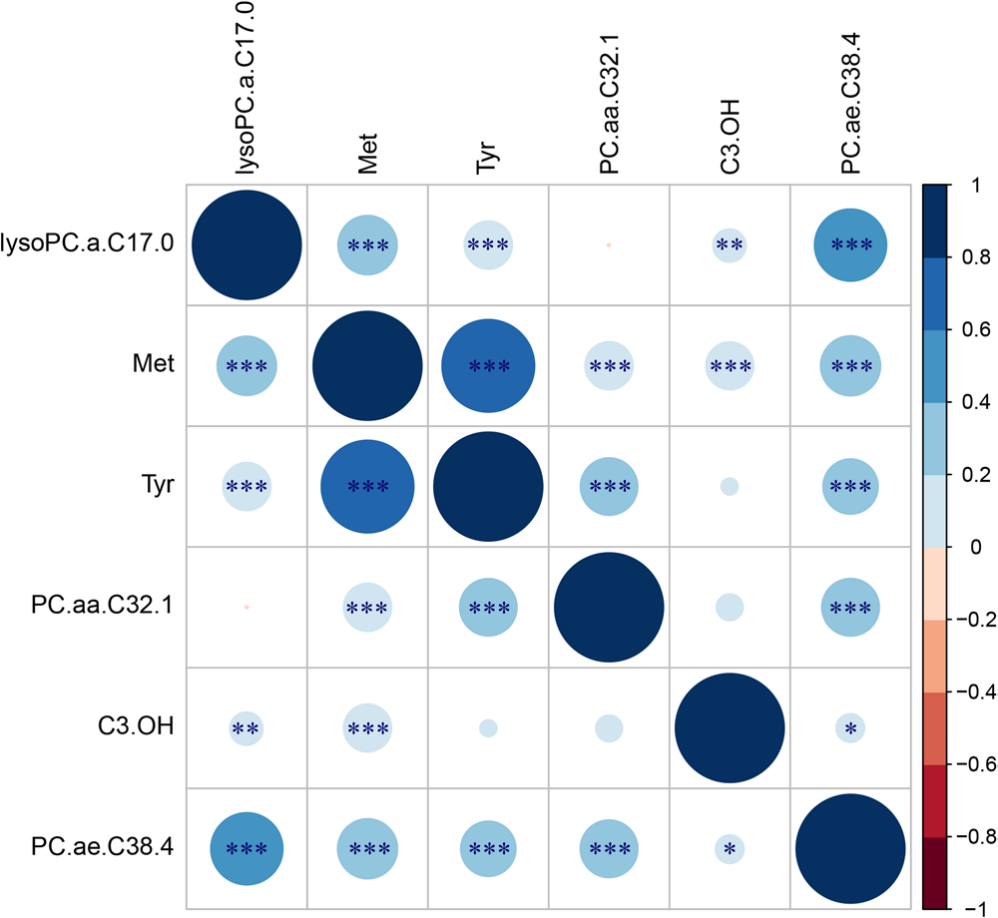
Correlogram of the six metabolites associated with LTL after correction for multiple testing in the first model using ERF data. Positive correlations are displayed in blue and negative correlations in red. Color intensity and the size of the circle are proportional to the correlation coefficients, with larger circles indicating higher correlation point estimates. Abbreviations: lysoPC.a.C17.0 = lysophosphatidylcholine acyl C17:0; Met = Methionine; Tyr = Tyrosine; PC.aa.C32.1 = phosphatidylcholine diacyl C32:1; C3.OH = hydroxypropionylcarnitine; PC.ae.C38.4 = phosphatidylcholine acyl-alkyl C38:4. *p-value < 0.05; **p-value < 0.01; ***p-value < 0.001.

To explore to which extent the various metabolites cluster, we constructed a heat map based on the correlation of each individual metabolite with LTL in both model 1 and model 2 (Fig. 1). The heat map shows two clusters of which one (hereafter referred to as “cluster 1”) includes lysoPC a C17:0, PC ae C38:4, and a series of PC ae metabolites positively associated with LTL, while the second cluster (hereafter referred to as “cluster 2”) includes methionine, tyrosine, PC aa C32:1, and a series of PC aa metabolites negatively associated with LTL. Figure 1 further shows that C3-OH is relatively dissimilar from all other metabolites. A correlogram of the six metabolites associated with LTL after correction for multiple testing is presented in Fig. 2 and shows a positive correlation of methionine with the three PC metabolites. LysoPC a C17:0 and PC ae C38:4 (cluster 1) are most strongly correlated in Fig. 2. Methionine and tyrosine are highly correlated with each other and both amino acids are correlated to PC aa C32.1 (Fig. 2).

**Figure 3:**
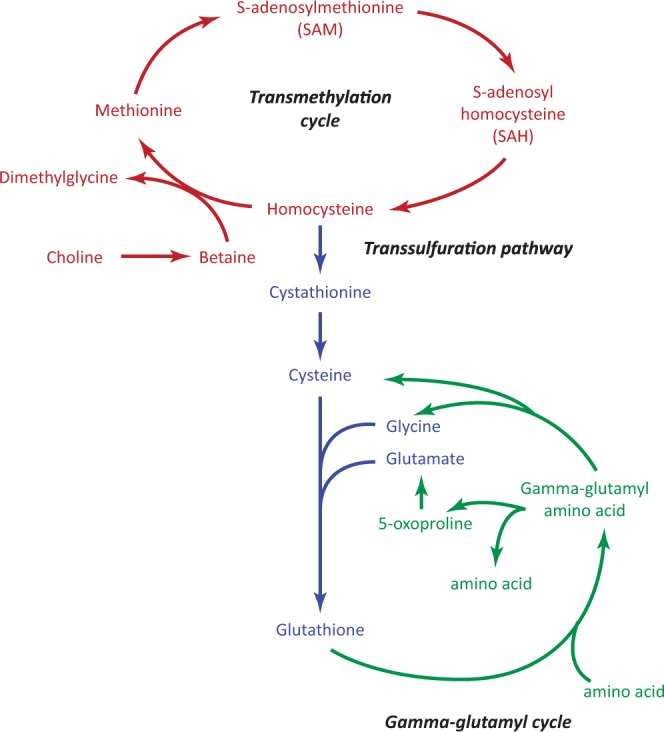
Methionine metabolic pathways. Indicated in red are the metabolites of the transmethylation pathway, in blue those of the transsulfuration pathway, and in green those involved in the gamma-glutamyl cycle. Modified figure from Dash *et al*.^113^.

Pathway analysis using the Taverna workflow showed that phosphatidylcholines (lysoPC a C17:0, PC aa C32:1, and PC ae C38:4) and methionine are involved in homocysteine metabolism. Homocysteine is the intermediate product in the conversion of the amino acid methionine to cysteine, a precursor of the antioxidant glutathione (Fig. 3). Briefly, PC is a precursor of choline which is oxidized to betaine. Betaine is used to convert homocysteine to methionine. Methionine is first converted to S-adenosylmethionine followed by demethylation to S-adenosylhomocysteine (SAH). Next, hydrolysis of SAH forms homocysteine, which can either be re-methylated into methionine (transmethylation cycle) or metabolized to cysteine (transsulfuration pathway) as shown in Fig. 3.

## Discussion

When adjusting for false positive findings due to multiple testing, this study identified significant associations between LTL and six metabolites, which form two extended clusters. These metabolites include three phosphatidylcholines (lysoPC a C17:0, PC aa C32:1, PC ae C38:4), two amino acids (methionine, tyrosine), and one acylcarnitine (C3-OH). Longer LTL was associated with higher levels of lysoPC a C17:0 and PC ae C38:4, and with lower levels of methionine, tyrosine, PC aa C32:1, and C3-OH. Pathway analysis highlighted a key role of the homocysteine metabolism.

A problem when comparing our findings to those in earlier studies is that the metabolites are platform-specific and thus we cannot replicate directly previous findings^43,44^. However, the PCs significantly associated with LTL in our study belong to the same metabolite family of glycerophospholipids^52^ as two metabolites significantly associated with LTL in the study of Zierer *et al*.^47^: 1‐stearoylglycerophosphoinositol and 1‐palmitoylglycero-phosphoinositol. These metabolites are involved in fatty acid metabolism and particularly membrane composition in biological aging. The small study (N = 423) performed in American Indians from the Strong Family Heart Study also found associations of glycerophospholipids (e.g. glycerophosphoethanolamines, glycero-phosphocholine, and glycerophosphoglycerol) with LTL^46^.

The metabolite most significantly associated with LTL in our study was lysoPC a C17:0. LysoPCs are formed through hydrolysis of PCs by phospholipase A2^53^ and have pro-atherogenic and pro-inflammatory effects through impairment of endothelium-dependent vascular relaxation^54^, monocyte recruitment and macrophage proliferation^55,56^, and increased expression of adhesion molecules^57^. Previously, negative associations of lysoPC a C17:0 levels with high-sensitivity C-reactive protein (hsCRP), interleukin-6, insulin, and myocardial infarction have been found^58,59^. These results indicate the involvement of inflammation^58,59^. Inflammation and cardiovascular disease are related to telomere shortening^60,61^ and our study brings to surface lysoPC a C17:0 as a novel key player.

We further identified a negative association of the two highly correlated amino acids methionine and tyrosine with LTL. Methionine is an essential amino acid and involved in multiple important biological processes necessary for normal growth and development in mammals, including protein synthesis, methylation, the transsulfuration pathway, and homocysteine metabolism^62^. Previous studies have shown that a methionine-restricted diet increased lifespan in rodents^63–67^. Tyrosine is a non-essential amino acid and a precursor for several catecholamines, including dopamine, as well as thyroid hormones (T_3_ and T_4_)^68,69^. Low thyroid hormone levels have been associated with increased lifespan in multiple animal models^70–72^ and in humans^73–75^. Moreover, a role of tyrosine as developmental regulator and modulator of longevity has been described in *Caenorhabditis elegans*^76^. Tyrosine can also form a dipeptide with gamma-glutamate called gamma-glutamyltyrosine (http://www.hmdb.ca/metabolites/HMDB0011741), which was negatively associated with LTL in the TwinsUk cohort^47^. It is involved in the gamma-glutamyl cycle (as shown in Fig. 3) and indicates involvement of increased oxidative stress^47^, a factor related to LTL shortening^77,78^. Elevated blood levels of the amino acid tyrosine are seen in obese individuals^79,80^, and were found to be a novel risk factor for the development of diabetes^38,81^. Type 2 diabetes has been associated with shorter LTL^10,16,17^.

Both methionine and tyrosine are correlated to PC aa C32:1, which is the fourth metabolite significantly associated with LTL. PCs are the major phospholipids in cell membranes and lipoproteins^82,83^. They consist of a glycerol backbone with different fatty acid combinations that are linked by ester (a) or ether (e) bonds, resulting in either diacyl (aa) or acyl-alkyl (ae) PCs^84^. We observed a cluster of metabolites (cluster 2) negatively associated with LTL, including methionine, tyrosine, PC aa C32:1 and multiple other diacyl PCs. Various metabolites of this cluster including PC aa C32:1, PC aa C36:1, PC aa C38:3, and PC aa C40:5, have been associated with increased risk of type 2 diabetes^40^. The other PC that surpassed the significance threshold in model 1 was PC ae C38:4. However, PC ae C38:4 was nominally significant after including BMI in the model (model 2) and after excluding the younger QIMR study from the meta-analysis. PC ae C38:4 showed a positive association with LTL and clustered with lysoPC a C17:0 and a series of PC ae metabolites (cluster 1) that also show consistent effect across cohorts such as PC ae C40:3, PC ae C40:4, and PC ae C40:5 (FDR < 0.05). In line with this finding, PC ae’s have been shown to have antioxidant properties, protecting lipids from oxidation^85,86^, and the metabolites in this cluster showed a reduced risk of type 2 diabetes^40^.

Although also C3-OH was found to be associated with LTL when adjusting for multiple testing, the association with LTL was only observed in ERF and LLS. In the other five studies, this metabolite did not pass the quality control. Therefore, this finding and other findings based on data of two studies only, such as PC aa C38:1, should be interpreted with care and more research, including alternative assessments of these metabolites, is needed. C3-OH is a metabolite of interest for further investigation as it is an acylcarnitine and involved in lipid transport as well as lipid and fatty acid metabolism (http://www.hmdb.ca/metabolites/HMDB0013125). Carnitine is mainly absorbed from the diet but can also be synthesized from the amino acids lysine and methionine^87^ and is essential for energy metabolism as it transports fatty acids from the cytosol into the mitochondrion for β-oxidation^88,89^. Figure 2 shows indeed that C3-OH is correlated to methionine, as predicted^87^. Carnitine insufficiency has been implicated as a common trait of insulin-resistant states, including advanced age, genetic diabetes, and diet-induced obesity^90^. However, when clustering the correlations of the metabolites to LTL, we find that C3-OH does not cluster with other metabolites (Fig. 1).

Pathway analysis using the Taverna workflow revealed that both methionine and PCs are part of homocysteine metabolism. Our results give novel metabolic insights into the findings of previous studies that describe an increase in plasma homocysteine with age and shortening of LTL with increasing homocysteine levels^91,92^. Our study suggests that lysoPC a C17:0, PC aa C32:1, PC ae C38:4 as well as methionine and tyrosine are key metabolites in the link between the homocysteine pathway and telomere length. Homocysteine metabolism has been implicated in a wide range of age-related diseases, such as cardiovascular diseases^93,94^, dementia^95,96^, Alzheimer’s disease^96,97^, diabetes and its associated vascular complications^98–100^, and in mortality^101–104^. Taking together the findings of our study with that of previous studies, the endothelium may be the tissue of interest. There is substantial evidence that homocysteine and lysoPC are involved in endothelial dysfunction^77,78^ caused by inflammation and oxidative stress^105–109^. In cultured endothelial cells, homocysteine was also shown to accelerate telomere shortening and endothelial senescence^92,110^.

A major strength of this study is that both LTL and metabolites were measured centrally, using a standard protocol and blood samples taken at the same time point. Metabolite levels were quantified using the AbsoluteIDQ p150 kit (Biocrates Life Sciences, Innsbruck, Austria) that detects biologically relevant metabolites from four compound classes: acylcarnitines, amino acids, hexoses, and phosho- and sphingolipids. This method has been proven to be in conformance with FDA Guideline ‘Guidance for Industry—Bioanalytical Method Validation (May 2001)’^111^, which implies proof of reproducibility within a given error range. At the same time, measuring metabolites with this specific platform may be considered also a limitation of our study as other metabolites might also be related to LTL.

In conclusion, using data from 7,853 individuals from seven independent cohorts, we found longer LTL associated with higher levels of lysoPC a C17:0 and PC ae C38:4, and with lower levels of methionine, tyrosine, PC aa C32:1, and C3-OH. These metabolites form two clusters, one including lysoPC a C17:0, PC ae C38:4, and a series of PC ae metabolites positively associated with LTL, while the second cluster includes methionine, tyrosine, PC aa C32:1, and a series of PC aa metabolites. These metabolites have been implicated in cardiovascular disease and diabetes, two major drivers of morbidity and mortality. The functional role of these metabolites involves inflammation and oxidative stress. Our pathway analysis links the metabolites to homocysteine metabolism, a pathway linked to cardiovascular disease, diabetes and many other age-related diseases.

## Supplementary information

Supplementary Materials

## Data Availability

All results generated during this study are included in this published article and its Supplementary Materials. The datasets analysed for each individual cohort can be requested by contacting the responsible Principal Investigator. Because of restrictions based on privacy regulations and informed consent of the participants, data cannot be made freely available in a public repository. For the Rotterdam Study data, requests should be directed towards the management team of the Rotterdam Study (secretariat.epi@erasmusmc.nl), which has a protocol for approving data requests.
