# Supplementary material for: Metabolomics reveals a link between homocysteine and lipid metabolism and leukocyte telomere length: the ENGAGE consortium
Source: Sci Rep. 2019 Aug 12;9:11623. doi: 10.1038/s41598-019-47282-6 (PMC6690953; doi:10.1038/s41598-019-47282-6)
Supplement: Supplementary file 1 — Supplementary Materials [file 41598_2019_47282_MOESM1_ESM.pdf]

Supplementary Material

Metabolomics reveals a link between homocysteine and lipid metabolism and leukocyte telomere length: the ENGAGE consortium

Ashley van der Spek[1ǂ], Linda Broer[1,2ǂ], Harmen HM Draisma[3-5ǂ], René Pool[3,4,6ǂ], Eva Albrecht[7], Marian Beekman[8], Massimo Mangino[9,10], Mait Raag[11], Dale R. Nyholt[12], Harish K. Dharuri[13], Veryan Codd[14], Najaf Amin[1], Eco J.C. de Geus[3,4], Joris Deelen[8,15], Ayse Demirkan[1,13], Idil Yet[9,16], Krista Fischer[17,18], Toomas Haller[17], Anjali K. Henders[19], Aaron Isaacs[20], Sarah E. Medland[21], Grant W. Montgomery[21], Simon P. Mooijaart[22], Konstantin Strauch[7,23], H. Eka D. Suchiman[8], Anika A.M. Vaarhorst[8], Diana van Heemst[22], Rui Wang-Sattler[24], John B. Whitfield[21], Gonneke Willemsen[3,4], Margaret J. Wright[25], Nicholas G. Martin[21], Nilesh J. Samani[14], Andres Metspalu[17], P. Eline Slagboom[8], Tim D. Spector[9], Dorret I. Boomsma[3,4,6#], Cornelia M. van Duijn[1,26,27#*], Christian Gieger[7#]

1 Department of Epidemiology, Erasmus Medical Center, Rotterdam, The Netherlands.
2 Department of Internal Medicine, Erasmus Medical Center, Rotterdam, The Netherlands.
3 Department of Biological Psychology, VU University Amsterdam, Amsterdam, The Netherlands.
4 Amsterdam Public Health research institute, Amsterdam University Medical Centers, The Netherlands.
5 Section of Genomics of Common Disease, Imperial College London, Burlington Danes Building Room E301, Du Cane Road, London W12 0NN.
6 BBMRI-NL: Infrastructure for the Application of Metabolomics Technology in Epidemiology (RP4), The Netherlands.
7 Institute of Genetic Epidemiology, Helmholtz Zentrum München - German Research Center for Environmental Health, Neuherberg, Germany.
8 Molecular Epidemiology, Department of Biomedical Data Sciences, Leiden University Medical Center, Leiden, The Netherlands.
9 Department of Twin Research and Genetic Epidemiology, King's College London, London, UK.
10 NIHR Biomedical Research Centre at Guy’s and St Thomas’ Foundation Trust, London SE1 9RT, UK.
11 Institute of Family Medicine and Public Health, University of Tartu, Tartu, Estonia.
12 School of Biomedical Sciences, Faculty of Health, Institute of Health and Biomedical Innovation, Queensland University of Technology, Brisbane, QLD, Australia.
13 Department of Human Genetics, Leiden University Medical Center, Leiden, the Netherlands.
14 Department of Cardiovascular Sciences, University of Leicester and NIHR Leicester Biomedical Research Centre, Glenfield Hospital, Leicester.
15 Max Planck Institute for Biology of Ageing, Cologne, Germany.

16 Department of Bioinformatics, Institute of Health Sciences, Hacettepe University, 06100, Ankara, Turkey.
17 Estonian Genome Center, Institute of Genomics, University of Tartu, Tartu, Estonia.
18 Institute of Mathematics and Statistics, University of Tartu, Estonia.
19 The Institute for Molecular Biosciences, The University of Queensland, Brisbane, Australia.
20 CARIM School for Cardiovascular Diseases, Maastricht Centre for Systems Biology (MaCSBio), and Department of Biochemistry, Maastricht University, Maastricht, the Netherlands.
21 QIMR Berghofer Medical Research Institute, Brisbane, Australia.
22 Department of Gerontology and Geriatrics, Leiden University Medical Center, Leiden, The Netherlands.
23 Chair of Genetic Epidemiology, IBE, Faculty of Medicine, LMU Munich, Germany.
24 Research Unit of Molecular Epidemiology, Helmholtz Zentrum München - German Research Center for Environmental Health, Neuherberg, Germany.
25 Queensland Brain Institute, The University of Queensland, Brisbane, Australia.
26 Leiden Academic Centre for Drug Research, Leiden University, Leiden, Netherlands.
27 Nuffield Department of Population Health, University of Oxford, Oxford, UK.

[ǂ]These authors contributed equally to this manuscript (shared first)
[#]These authors contributed equally to this manuscript (shared last)

*Corresponding author:

Prof. Cornelia M. van Duijn
c.vanduijn@erasmusmc.nl
Department of Epidemiology
Erasmus Medical Center
P.O. Box 2040, 3000 CA
Rotterdam, the Netherlands

Cohort descriptions

The **KORA** (Cooperative Health Research in the Region of Augsburg) study is a series of independent population-based epidemiological surveys and follow-up studies of participants living in the region of Augsburg, Southern Germany[1]. All survey participants are of German nationality, identified through the registration office. Informed consent has been given by all participants. The present study includes data of the KORA F4 (2006-2008) study which is a follow-up study of the KORA S4 survey (1999-2001).

The Netherlands Twin Register (**NTR**: http://www.tweelingenregister.org/) recruits twins and their family members to study the causes of individual differences in health, behavior and lifestyle. Participants are followed longitudinally; details about the cohort have been published previously[2]. A subsample of unselected twins and their family members has taken part in the NTR-Biobank[3] in which biological samples, including DNA and RNA, were collected in a standardized manner after overnight fasting. Study protocols were approved by the Central Ethics Committee on Research Involving Human Subjects of the VU University Medical Centre, Amsterdam, an Institutional Review Board certified by the U.S. Office of Human Research Protections (IRB number IRB00002991 under Federal-wide Assurance- FWA00017598; IRB/institute codes, NTR 03-180).

The Estonian Genome Center, University of Tartu (**EGCUT**) is a population-based biobank of the Estonian Genome Project of University of Tartu (www.biobank.ee)[4]. The current cohort size is over 51,515, from 18 years of age and up, which reflects closely the age distribution in the adult Estonian population. The samples included in this study form a random subset of the cohort, with the exception of 500 female individuals aged 83+ which were specifically selected

according to age and sex. Subjects are recruited by the general practitioners (GP) and physicians in the hospitals. Each participant filled out a Computer Assisted Personal interview, including personal data (place of birth, place(s) of living, nationality etc.), genealogical data (family history, three generations), educational and occupational history and lifestyle data (physical activity, dietary habits, smoking, alcohol consumption, women's health, quality of life). Anthropometric and physiological measurements were also taken.

The UK Adult Twin Registry (or **TwinsUK** Registry) is a cohort of volunteer adult twins from all over the United Kingdom[5]. The Registry was started in 1992 with the primary aim of assessment of heritability of osteoarthritis and osteoporosis in women. The success of early studies led to rapid evolution of the registry and it now incorporates about 13 000 twins, both male and female aged 18–103 years. For a total of 1235 participants, samples have been measured using the Biocrates Absolute IDQ™-kit p150 (BIOCRATES Life Sciences AG, Innsbruck, Austria) metabolomics platform[5,6]. In total, 810 participants with both LTL qPCR measurements as well as metabolomics data (Biocrates) were included in this analysis. Ethical approval was obtained from the Guy's and St. Thomas' Hospital Ethics Committee. Written informed consent was obtained from every participant in the study.

The Erasmus Rucphen Family (**ERF**) study is a family-based study including 3,000 living descendants of 22 couples who had at least 6 children baptized in the community church around 1850-1900. Individuals who were 18 years or older were invited to participate in the study and were not selected on any disease or other outcome. Details about the genealogy of the population have been described elsewhere[7,8]. The study protocol was approved by the

Medical Ethical Committee of the Erasmus University Medical Center, Rotterdam, the Netherlands. All participants provided written informed consent.

For the Leiden Longevity Study (**LLS**), long-lived siblings of Dutch descent were recruited together with their offspring and the partners of thereof. Families were included if at least two long-lived siblings were alive and fulfilled the age criterion of 89 years or older for males and 91 years or older for females, representing less than 0.5% of the Dutch population in 2001[9]. In total, 944 long-lived proband siblings from 421 families with a mean age of 94 years (range, 89-104), 1,671 offspring (61 years, 39-81), and 744 partners (60 years, 36-79) were included in the study. DNA from the LLS was extracted from samples at baseline using conventional methods[10]. For the current analysis only the offspring and their partners were used.

The Queensland Institute of Medical Research (**QIMR**) adolescent study comprised twins and their non-twin siblings living in south-east Queensland, Australia[11]. Most (98% by self-report) are of mixed European ancestry, mainly from the British Isles. The participants are not selected on the basis of any disease or other outcome. Blood samples were collected at the end of testing sessions from participants and, if possible, from their parents. Pedigree relationships and zygosity were confirmed by genotype data. Further details are provided elsewhere[12].

Telomere length measurements

All samples from all studies were measured in the same laboratory under standard conditions. Mean leukocyte telomere length was measured by quantitative PCR-based technique as previously described[13,14]. This method expresses telomere length as a ratio (T/S) of telomere repeat length (T) to copy number of a single copy gene, 36B4(S), within each sample. Samples

were quantified relative to a calibrator sample used on each run (DNA from the K562 cell line)[14]. Mean inter-run coefficients of variation (CVs) were calculated for all study cohorts and these were less than 5% in all.

Metabolite measurements and quality control

Targeted metabolite profiling was performed on a targeted metabolomics platform (Biocrates AbsoluteIDQ p150 kit, BIOCRATES Life Sciences AG, Austria) using electrospray ionization tandem mass spectrometry (MS/MS). The company had no access to genotype or phenotype information that would have permitted any data pre-filtering other than objective quality control for measurement errors based on internal controls and duplicates. Details of the metabolomics measurement technique are described by patent US 2007/0004044 (accessible online at http://www.freepatentsonline.com/20070004044.html). A summary of the method can be found in [15,16] and an overview of the metabolomics field and the related technologies is provided in the review paper by Wenk[17]. In summary, a targeted profiling scheme is used to quantitatively screen for known small-molecule metabolites using multiple reaction monitoring, neutral loss and precursor ion scans. Quantification of the metabolites in the biological sample is achieved by reference to appropriate internal standards. The reproducibility of the Biocrates kit was determined by assaying three spiking levels of blood plasma (low, medium, high) using the available standards with six replicates.

Metabolite quality control was performed separately per study and has been described previously[18]. A summary is provided below. CVs were calculated for each metabolite i and plate

j, as metabolite profile measurements were performed on multiple plates: $CV_{i,j} = \frac{sd_{i,j}}{mean_{i,j}}$,

where the standard deviation (*sd)* and *mean* were calculated over all five reference measurements per plate *j*. For each cohort, the summary statistics were compared with measurement detection limit specifications provided by BIOCRATES Life Sciences AG.

There were three reasons to exclude a metabolite from analyses (Supplementary Table 1): the mean CV_i over all plates was higher than 25% and/or there were more than 5% missing values. Additionally, metabolites reported as absolute concentrations (µM) were excluded if the median was below the lower limit of quantification (LLOQ) and semi-quantitatively determined metabolites were excluded if their median measurement values were below the limit of detection (LOD). Outlying metabolite concentration values (data points) were also excluded. Outlying data points were defined as data points that deviated more than five SDs from the mean value for each metabolite. Samples were excluded if they contained more than three outlying data points in metabolites that were less than 70% correlated with each other. Missing values in the remaining metabolite data were imputed using a multiple imputation procedure as implemented in the 'mice' package in the statistical language and environment "R"[19].

Supplementary references

1 Wichmann, H. E., Gieger, C., Illig, T. & Group, M. K. S. KORA-gen--resource for population genetics, controls and a broad spectrum of disease phenotypes. *Gesundheitswesen* **67 Suppl 1**, S26-30, doi:10.1055/s-2005-858226 (2005).

2 Boomsma, D. I. *et al.* Netherlands Twin Register: from twins to twin families. *Twin Res Hum Genet* **9**, 849-857, doi:10.1375/183242706779462426 (2006).

3 Willemsen, G. *et al.* The Netherlands Twin Register biobank: a resource for genetic epidemiological studies. *Twin Res Hum Genet* **13**, 231-245, doi:10.1375/twin.13.3.231 (2010).

4 Nelis, M. *et al.* Genetic structure of Europeans: a view from the North-East. *PLoS One* **4**, e5472, doi:10.1371/journal.pone.0005472 (2009).

5 Moayyeri, A., Hammond, C. J., Valdes, A. M. & Spector, T. D. Cohort Profile: TwinsUK and healthy ageing twin study. *Int. J. Epidemiol.* **42**, 76-85, doi:10.1093/ije/dyr207 (2013).

6 Menni, C. *et al.* Targeted metabolomics profiles are strongly correlated with nutritional patterns in women. *Metabolomics* **9**, 506-514, doi:10.1007/s11306-012-0469-6 (2013).

7 Aulchenko, Y. S. *et al.* Linkage disequilibrium in young genetically isolated Dutch population. *Eur. J. Hum. Genet.* **12**, 527-534, doi:10.1038/sj.ejhg.5201188 (2004).

8 Pardo, L. M., MacKay, I., Oostra, B., van Duijn, C. M. & Aulchenko, Y. S. The effect of genetic drift in a young genetically isolated population. *Ann. Hum. Genet.* **69**, 288-295, doi:10.1046/j.1529-8817.2005.00162.x (2005).

9 Schoenmaker, M. *et al.* Evidence of genetic enrichment for exceptional survival using a family approach: the Leiden Longevity Study. *Eur. J. Hum. Genet.* **14**, 79-84, doi:10.1038/sj.ejhg.5201508 (2006).

10 Beekman, M. *et al.* Chromosome 4q25, microsomal transfer protein gene, and human longevity: novel data and a meta-analysis of association studies. *J. Gerontol. A Biol. Sci. Med. Sci.* **61**, 355-362 (2006).

11 Wright, M. J. & Martin, N. G. Brisbane adolescent twin study: outline of study methods and research projects. *Aust J Psychol* **56**, 65-78 (2004).

12 Medland, S. E. *et al.* Common variants in the trichohyalin gene are associated with straight hair in Europeans. *Am. J. Hum. Genet.* **85**, 750-755, doi:10.1016/j.ajhg.2009.10.009 (2009).

13 Cawthon, R. M. Telomere measurement by quantitative PCR. *Nucleic Acids Res.* **30**, e47 (2002).

14 Codd, V. *et al.* Common variants near TERC are associated with mean telomere length. *Nat. Genet.* **42**, 197-199, doi:10.1038/ng.532 (2010).

15 Unterwurzacher, I., Koal, T., Bonn, G. K., Weinberger, K. M. & Ramsay, S. L. Rapid sample preparation and simultaneous quantitation of prostaglandins and lipoxygenase derived fatty acid metabolites by liquid chromatography-mass spectrometry from small sample volumes. *Clin. Chem. Lab. Med.* **46**, 1589-1597, doi:10.1515/CCLM.2008.323 (2008).

16 Weinberger, K. M. [Metabolomics in diagnosing metabolic diseases] Einsatz von Metabolomics zur Diagnose von Stoffwechselkrankheiten. *Ther. Umsch.* **65**, 487-491, doi:10.1024/0040-5930.65.9.487 (2008).

17 Wenk, M. R. The emerging field of lipidomics. *Nat Rev Drug Discov* **4**, 594-610, doi:10.1038/nrd1776 (2005).

18 Draisma, H. H. M. *et al.* Genome-wide association study identifies novel genetic variants contributing to variation in blood metabolite levels. *Nat Commun* **6**, 7208, doi:10.1038/ncomms8208 (2015).

19 R: A language and environment for statistical computing v. 2.12.1 (R foundation for Statistical Computing, Vienna, Austria, 2010).

Supplementary Tables

Supplementary Table 1: Exclusion reasons for each metabolite per cohort

	KORA			NTR			EGCUT			TwinsUK			ERF			LLS			QIMR		
**Mtb**	**C1**	**C2**	**C3**	**C1**	**C2**	**C3**	**C1**	**C2**	**C3**	**C1**	**C2**	**C3**	**C1**	**C2**	**C3**	**C1**	**C2**	**C3**	**C1**	**C2**	**C3**
C0	+	+	+	+	+	+	+	+	+	+	+	+	+	+	+	+	+	+	+	+	+
C10	+	+	+	+	+	-	-	+	-	+	+	-	+	+	+	+	+	-	+	+	-
C10:1	+	+	+	+	+	+	-	+	-	+	+	+	+	+	+	+	+	+	+	+	-
C10:2	+	+	+	+	+	+	-	+	-	+	+	+	+	+	+	+	+	+	+	+	-
C12	+	+	-	+	+	-	-	+	-	+	+	-	+	+	-	+	+	-	+	+	-
C12-DC	+	+	-	+	+	-	-	+	-	+	+	-	+	+	-	+	+	-	+	+	-
C12:1	+	+	+	+	+	+	+	+	+	+	+	+	+	+	+	+	+	+	+	+	+
C14	+	+	-	+	+	-	-	+	-	+	+	-	+	+	-	+	+	-	+	+	-
C14:1	+	+	+	+	+	+	+	+	+	+	+	+	+	+	+	+	+	+	+	+	+
C14:1-OH	+	+	-	+	+	-	-	+	-	+	+	+	+	+	-	+	+	+	+	+	-
C14:2	+	+	+	+	+	+	-	+	-	+	+	+	+	+	+	+	+	+	+	+	-
C14:2-OH	+	+	-	+	+	-	-	+	-	+	+	-	+	+	-	+	+	-	+	+	-
C16	+	+	-	+	+	-	+	+	-	+	+	-	+	+	-	+	+	-	+	+	-
C16-OH	-	+	-	-	+	-	-	+	-	-	+	-	+	+	-	+	+	-	+	+	-
C16:1	+	+	-	+	+	-	-	+	-	+	+	-	+	+	-	+	+	-	+	+	-
C16:1-OH	+	+	-	-	+	-	-	+	-	+	+	-	+	+	-	+	+	-	+	+	-
C16:2	-	+	-	-	+	-	-	+	-	-	+	+	+	+	+	+	+	-	+	+	-
C16:2-OH	+	+	-	-	+	-	-	+	-	+	+	-	-	+	-	+	+	-	+	+	-
C18	+	+	-	+	+	-	-	+	-	+	+	-	+	+	-	+	+	-	+	+	-
C18:1	+	+	+	+	+	+	+	+	-	+	+	+	+	+	+	+	+	+	+	+	+
C18:1-OH	-	+	-	-	+	-	-	+	-	-	+	-	+	+	-	+	+	-	+	+	-
C18:2	+	+	+	+	+	+	+	+	+	+	+	+	+	+	+	+	+	+	+	+	+
C2	+	+	+	+	+	+	+	+	+	+	+	+	+	+	+	+	+	+	+	+	+
C3	+	+	-	+	+	-	+	+	-	+	+	-	+	+	+	+	+	+	+	+	-
C3-DC (C4-OH)	+	+	-	+	+	-	-	+	-	+	+	-	+	+	-	+	+	-	+	+	-
C3-OH	-	+	-	-	+	-	-	+	-	-	+	-	+	+	+	+	+	+	+	+	-
C3:1	-	+	-	-	+	-	-	+	-	-	+	-	+	+	-	+	+	-	+	+	-
C4	+	+	-	+	+	-	+	+	-	+	+	-	+	+	-	+	+	-	+	+	-
C4:1	-	+	-	+	+	-	-	+	-	-	+	-	+	+	-	+	+	-	+	+	-
C5	+	+	-	+	+	-	+	+	-	+	+	-	+	+	-	+	+	-	+	+	-
C5-DC (C6-OH)	+	+	-	+	+	-	-	+	-	+	+	-	+	+	-	+	+	-	+	+	-
C5-M-DC	+	+	-	+	+	-	-	+	-	+	+	-	+	+	-	+	+	-	+	+	-
C5-OH (C3-DC-M)	+	+	-	+	+	-	-	+	-	+	+	-	+	+	-	+	+	-	+	+	-
C5:1	+	+	-	+	+	-	-	+	-	+	+	-	+	+	-	+	+	-	+	+	-
C5:1-DC	+	+	-	+	+	+	-	+	-	+	+	-	+	+	+	+	+	-	+	+	-

	KORA			NTR			EGCUT			TwinsUK			ERF			LLS			QIMR		
**Mtb**	**C1**	**C2**	**C3**	**C1**	**C2**	**C3**	**C1**	**C2**	**C3**	**C1**	**C2**	**C3**	**C1**	**C2**	**C3**	**C1**	**C2**	**C3**	**C1**	**C2**	**C3**
C6 (C4:1-DC)	+	+	-	+	+	-	-	+	-	+	+	-	+	+	-	+	+	-	+	+	-
C6:1	+	+	-	+	+	-	-	+	-	+	+	-	+	+	-	+	+	-	+	+	-
C7-DC	+	+	+	+	+	+	-	+	-	+	+	+	+	+	+	+	+	+	+	+	-
C8	+	+	+	+	+	-	-	+	-	+	+	-	+	+	+	+	+	-	+	+	-
C8:1	+	+	+	+	+	+	+	+	+	+	+	+	+	+	+	+	+	+	+	+	+
C9	+	+	+	+	+	-	-	+	-	+	+	+	+	+	+	+	+	+	+	+	-
Arg	+	+	+	+	+	+	+	+	-	+	+	+	+	+	+	+	+	+	+	+	-
Gln	+	+	+	+	+	+	+	+	+	+	+	+	+	+	+	+	+	+	+	+	+
Gly	+	+	+	+	+	+	+	+	+	+	+	+	+	+	+	+	+	+	+	+	+
His	+	+	+	+	+	+	+	+	+	+	+	+	+	+	+	+	+	+	+	+	+
Met	+	+	+	+	+	+	+	+	+	+	+	+	+	+	+	+	+	+	+	+	+
Orn	+	+	+	+	+	+	+	+	+	+	+	+	+	+	+	+	+	+	+	+	+
Phe	+	+	+	+	+	+	+	+	+	+	+	+	+	+	+	+	+	+	+	+	+
Pro	+	+	+	+	+	+	+	+	+	+	+	+	+	+	+	+	+	+	+	+	+
Ser	+	+	+	+	+	+	+	+	+	+	+	+	+	+	+	+	+	+	+	+	+
Thr	+	+	+	+	+	+	+	+	-	+	+	+	-	+	+	+	+	+	+	+	+
Trp	+	+	+	+	+	+	+	+	-	+	+	+	-	+	+	+	+	+	+	+	+
Tyr	+	+	+	+	+	+	+	+	+	+	+	+	-	+	+	+	+	+	+	+	+
Val	+	+	+	+	+	+	+	+	+	+	+	+	-	+	+	+	+	+	+	+	+
xLeu	+	+	+	+	+	+	+	+	+	+	+	+	-	+	+	+	+	+	+	+	+
PC aa C24:0	+	+	+	-	+	+	-	+	-	+	+	+	+	+	-	+	+	-	+	+	+
PC aa C26:0	+	+	-	+	+	-	-	+	-	+	+	-	+	+	-	+	+	-	+	+	-
PC aa C28:1	+	+	+	+	+	+	+	+	+	+	+	+	+	+	+	+	+	+	+	+	+
PC aa C30:0	+	+	+	+	+	+	+	+	+	+	+	+	+	+	+	+	+	+	+	+	+
PC aa C30:2	-	+	-	-	-	-	-	-	+	-	+	+	-	-	-	-	+	-	+	-	+
PC aa C32:0	+	+	+	+	+	+	+	+	+	+	+	+	+	+	+	+	+	+	+	+	+
PC aa C32:1	+	+	+	+	+	+	+	+	+	+	+	+	+	+	+	+	+	+	+	+	+
PC aa C32:2	+	+	+	+	+	+	+	+	+	+	+	+	+	+	+	+	+	+	+	+	+
PC aa C32:3	+	+	+	+	+	+	+	+	+	+	+	+	+	+	+	+	+	+	+	+	+
PC aa C34:1	+	+	+	+	+	+	+	+	+	+	+	+	+	+	+	+	+	+	+	+	+
PC aa C34:2	+	+	+	+	+	+	+	+	+	+	+	+	+	+	+	+	+	+	+	+	+
PC aa C34:3	+	+	+	+	+	+	+	+	+	+	+	+	+	+	+	+	+	+	+	+	+
PC aa C34:4	+	+	+	+	+	+	+	+	+	+	+	+	+	+	+	+	+	+	+	+	+
PC aa C36:0	+	+	+	+	+	+	+	+	+	+	+	+	+	+	+	+	+	+	+	+	+
PC aa C36:1	+	+	+	+	+	+	+	+	+	+	+	+	+	+	+	+	+	+	+	+	+
PC aa C36:2	+	+	+	+	+	+	+	+	+	+	+	+	+	+	+	+	+	+	+	+	+
PC aa C36:3	+	+	+	+	+	+	+	+	+	+	+	+	+	+	+	+	+	+	+	+	+
PC aa C36:4	+	+	+	+	+	+	+	+	+	+	+	+	+	+	+	+	+	+	+	+	+
PC aa C36:5	+	+	+	+	+	+	+	+	+	+	+	+	+	+	+	+	+	+	+	+	+
PC aa C36:6	+	+	+	+	+	+	+	+	+	+	+	+	+	+	+	+	+	+	+	+	+

	KORA			NTR			EGCUT			TwinsUK			ERF			LLS			QIMR		
**Mtb**	**C1**	**C2**	**C3**	**C1**	**C2**	**C3**	**C1**	**C2**	**C3**	**C1**	**C2**	**C3**	**C1**	**C2**	**C3**	**C1**	**C2**	**C3**	**C1**	**C2**	**C3**
PC aa C38:0	+	+	+	+	+	+	+	+	+	+	+	+	+	+	+	+	+	+	+	+	+
PC aa C38:1	-	+	-	-	+	+	-	+	+	-	+	+	-	+	+	+	+	+	+	+	+
PC aa C38:3	+	+	+	+	+	+	+	+	+	+	+	+	+	+	+	+	+	+	+	+	+
PC aa C38:4	+	+	+	+	+	+	+	+	+	+	+	+	+	+	+	+	+	+	+	+	+
PC aa C38:5	+	+	+	+	+	+	+	+	+	+	+	+	+	+	+	+	+	+	+	+	+
PC aa C38:6	+	+	+	+	+	+	+	+	+	+	+	+	+	+	+	+	+	+	+	+	+
PC aa C40:1	+	+	+	+	+	+	+	+	-	+	+	+	+	+	-	+	+	+	+	+	-
PC aa C40:2	+	+	+	+	+	+	+	+	+	+	+	+	+	+	+	+	+	+	+	+	+
PC aa C40:3	+	+	+	+	+	+	+	+	+	+	+	+	+	+	+	+	+	+	+	+	+
PC aa C40:4	+	+	+	+	+	+	+	+	+	+	+	+	+	+	+	+	+	+	+	+	+
PC aa C40:5	+	+	+	+	+	+	+	+	+	+	+	+	+	+	+	+	+	+	+	+	+
PC aa C40:6	+	+	+	+	+	+	+	+	+	+	+	+	+	+	+	+	+	+	+	+	+
PC aa C42:0	+	+	+	+	+	+	+	+	+	+	+	+	+	+	+	+	+	+	+	+	+
PC aa C42:1	+	+	+	+	+	+	+	+	+	+	+	+	+	+	+	+	+	+	+	+	+
PC aa C42:2	+	+	+	+	+	+	+	+	+	+	+	+	+	+	+	+	+	+	+	+	+
PC aa C42:4	+	+	+	+	+	+	+	+	+	+	+	+	+	+	+	+	+	+	+	+	+
PC aa C42:5	+	+	+	+	+	+	+	+	+	+	+	+	+	+	+	+	+	+	+	+	+
PC aa C42:6	+	+	+	+	+	+	+	+	-	+	+	+	+	+	+	+	+	+	+	+	+
PC ae C30:0	+	+	+	+	+	+	+	+	-	+	+	+	+	+	+	+	+	+	+	+	+
PC ae C30:1	-	+	-	-	+	+	-	-	+	-	+	+	-	-	+	-	+	-	+	-	+
PC ae C30:2	+	+	-	+	+	-	-	+	-	+	+	-	+	+	-	+	+	-	+	+	-
PC ae C32:1	+	+	+	+	+	+	+	+	+	+	+	+	+	+	+	+	+	+	+	+	+
PC ae C32:2	+	+	+	+	+	+	+	+	+	+	+	+	+	+	+	+	+	+	+	+	+
PC ae C34:0	+	+	+	+	+	+	+	+	+	+	+	+	+	+	+	+	+	+	+	+	+
PC ae C34:1	+	+	+	+	+	+	+	+	+	+	+	+	+	+	+	+	+	+	+	+	+
PC ae C34:2	+	+	+	+	+	+	+	+	+	+	+	+	+	+	+	+	+	+	+	+	+
PC ae C34:3	+	+	+	+	+	+	+	+	+	+	+	+	+	+	+	+	+	+	+	+	+
PC ae C36:0	+	+	+	+	+	+	+	+	+	+	+	+	+	+	+	+	+	+	+	+	+
PC ae C36:1	+	+	+	+	+	+	+	+	+	+	+	+	+	+	+	+	+	+	+	+	+
PC ae C36:2	+	+	+	+	+	+	+	+	+	+	+	+	+	+	+	+	+	+	+	+	+
PC ae C36:3	+	+	+	+	+	+	+	+	+	+	+	+	+	+	+	+	+	+	+	+	+
PC ae C36:4	+	+	+	+	+	+	+	+	+	+	+	+	+	+	+	+	+	+	+	+	+
PC ae C36:5	+	+	+	+	+	+	+	+	+	+	+	+	+	+	+	+	+	+	+	+	+
PC ae C38:0	+	+	+	+	+	+	+	+	+	+	+	+	+	+	+	+	+	+	+	+	+
PC ae C38:1	+	+	+	+	+	+	+	+	+	+	+	+	-	+	+	+	+	+	+	+	+
PC ae C38:2	+	+	+	+	+	+	+	+	+	+	+	+	+	+	+	+	+	+	+	+	+
PC ae C38:3	+	+	+	+	+	+	+	+	+	+	+	+	+	+	+	+	+	+	+	+	+
PC ae C38:4	+	+	+	+	+	+	+	+	+	+	+	+	+	+	+	+	+	+	+	+	+
PC ae C38:5	+	+	+	+	+	+	+	+	+	+	+	+	+	+	+	+	+	+	+	+	+
PC ae C38:6	+	+	+	+	+	+	+	+	+	+	+	+	+	+	+	+	+	+	+	+	+

	KORA			NTR			EGCUT			TwinsUK			ERF			LLS			QIMR		
**Mtb**	**C1**	**C2**	**C3**	**C1**	**C2**	**C3**	**C1**	**C2**	**C3**	**C1**	**C2**	**C3**	**C1**	**C2**	**C3**	**C1**	**C2**	**C3**	**C1**	**C2**	**C3**
PC ae C40:0	+	+	-	+	+	-	-	+	-	+	+	-	+	+	-	+	+	-	+	+	-
PC ae C40:1	+	+	+	+	+	+	+	+	+	+	+	+	+	+	+	+	+	+	+	+	+
PC ae C40:2	+	+	+	+	+	+	+	+	+	+	+	+	+	+	+	+	+	+	+	+	+
PC ae C40:3	+	+	+	+	+	+	+	+	+	+	+	+	+	+	+	+	+	+	+	+	+
PC ae C40:4	+	+	+	+	+	+	+	+	+	+	+	+	+	+	+	+	+	+	+	+	+
PC ae C40:5	+	+	+	+	+	+	+	+	+	+	+	+	+	+	+	+	+	+	+	+	+
PC ae C40:6	+	+	+	+	+	+	+	+	+	+	+	+	+	+	+	+	+	+	+	+	+
PC ae C42:0	+	+	+	+	+	+	+	+	-	+	+	+	+	+	+	+	+	+	+	+	+
PC ae C42:1	+	+	+	+	+	+	+	+	+	+	+	+	+	+	+	+	+	+	+	+	+
PC ae C42:2	+	+	+	+	+	+	+	+	+	+	+	+	+	+	+	+	+	+	+	+	+
PC ae C42:3	+	+	+	+	+	+	+	+	+	+	+	+	+	+	+	+	+	+	+	+	+
PC ae C42:4	+	+	+	+	+	+	+	+	+	+	+	+	+	+	+	+	+	+	+	+	+
PC ae C42:5	+	+	+	+	+	+	+	+	-	+	+	+	+	+	+	+	+	+	+	+	+
PC ae C44:3	+	+	+	+	+	+	+	+	+	+	+	+	+	+	+	+	+	+	+	+	+
PC ae C44:4	+	+	+	+	+	+	+	+	+	+	+	+	+	+	+	+	+	+	+	+	+
PC ae C44:5	+	+	+	+	+	+	+	+	+	+	+	+	+	+	+	+	+	+	+	+	+
PC ae C44:6	+	+	+	+	+	+	+	+	+	+	+	+	+	+	+	+	+	+	+	+	+
lysoPC a C14:0	+	+	-	+	+	-	-	+	-	+	+	-	+	+	-	+	+	-	+	+	-
lysoPC a C16:0	+	+	+	+	+	+	+	+	+	+	+	+	+	+	+	+	+	+	+	+	+
lysoPC a C16:1	+	+	+	+	+	+	+	+	+	+	+	+	+	+	+	+	+	+	+	+	+
lysoPC a C17:0	+	+	+	+	+	+	+	+	+	+	+	+	+	+	+	+	+	+	+	+	+
lysoPC a C18:0	+	+	+	+	+	+	+	+	+	+	+	+	+	+	+	+	+	+	+	+	+
lysoPC a C18:1	+	+	+	+	+	+	+	+	+	+	+	+	+	+	+	+	+	+	+	+	+
lysoPC a C18:2	+	+	+	+	+	+	+	+	+	+	+	+	+	+	+	+	+	+	+	+	+
lysoPC a C20:3	+	+	+	+	+	+	+	+	+	+	+	+	+	+	+	+	+	+	+	+	+
lysoPC a C20:4	+	+	+	+	+	+	+	+	+	+	+	+	+	+	+	+	+	+	+	+	+
lysoPC a C24:0	+	+	-	+	+	-	-	+	-	+	+	-	+	+	-	+	+	-	+	+	-
lysoPC a C26:0	-	+	-	+	+	+	-	+	-	-	+	-	+	+	-	+	+	-	+	+	-
lysoPC a C26:1	+	+	-	+	+	-	-	+	-	+	+	-	+	+	-	+	+	-	+	+	-
lysoPC a C28:0	+	+	+	+	+	+	+	+	-	+	+	+	+	+	+	+	+	+	+	+	+
lysoPC a C28:1	+	+	+	+	+	+	+	+	+	+	+	+	+	+	+	+	+	+	+	+	+
lysoPC a C6:0	-	+	-	-	+	-	-	-	-	-	+	-	-	-	-	-	+	-	+	-	-
SM (OH) C14:1	+	+	+	+	+	+	+	+	+	+	+	+	+	+	+	+	+	+	+	+	+
SM (OH) C16:1	+	+	+	+	+	+	+	+	+	+	+	+	-	+	+	+	+	+	+	+	+
SM (OH) C22:1	+	+	+	+	+	+	+	+	+	+	+	+	-	+	+	+	+	+	+	+	+
SM (OH) C22:2	+	+	+	+	+	+	+	+	+	+	+	+	-	+	+	+	+	+	+	+	+
SM (OH) C24:1	+	+	+	+	+	+	+	+	+	+	+	+	-	+	+	+	+	+	+	+	+
SM C16:0	+	+	+	+	+	+	+	+	+	+	+	+	+	+	+	+	+	+	+	+	+
SM C16:1	+	+	+	+	+	+	+	+	+	+	+	+	+	+	+	+	+	+	+	+	+
SM C18:0	+	+	+	+	+	+	+	+	+	+	+	+	+	+	+	+	+	+	+	+	+

	KORA			NTR			EGCUT			TwinsUK			ERF			LLS			QIMR		
**Mtb**	**C1**	**C2**	**C3**	**C1**	**C2**	**C3**	**C1**	**C2**	**C3**	**C1**	**C2**	**C3**	**C1**	**C2**	**C3**	**C1**	**C2**	**C3**	**C1**	**C2**	**C3**
SM C18:1	+	+	+	+	+	+	+	+	+	+	+	+	+	+	+	+	+	+	+	+	+
SM C20:2	+	+	+	+	+	+	+	+	+	+	+	+	+	+	+	+	+	+	+	+	+
SM C22:3	-	+	-	-	-	-	-	-	-	-	+	+	-	-	-	-	+	-	+	-	-
SM C24:0	+	+	+	+	+	+	+	+	+	+	+	+	+	+	+	+	+	+	+	+	+
SM C24:1	+	+	+	+	+	+	+	+	+	+	+	+	+	+	+	+	+	+	+	+	+
SM C26:0	+	+	+	+	+	+	-	+	+	+	+	+	+	+	+	+	+	+	+	+	+
SM C26:1	+	+	+	+	+	+	+	+	+	+	+	+	+	+	+	+	+	+	+	+	+
H1	+	+	+	+	+	+	+	+	+	+	+	+	+	+	+	+	+	+	+	+	+

C1: Median(Concentration) > LOD, where LOD is limit of detection specified by Biocrates
C2: Excluded based on local QC of the metabolomics data.
C3: f(0) < 5%, where f(0)=N(0)/Samplesize. For the Biocrates data set a value of exactly 0 is considered a missing value.
'+': passed QC for this criterion; '-': failed QC for this criterion

Supplementary Table 2: Complete partial correlation meta-analysis results of telomere length and metabolites

	Model 1: age + sex				Model 2: age + sex + BMI				
**metabolite**	**n**	**direction***	**r**	**p-value**	**n**	**direction***	**r**	**p-value**	**metabolite full name**
C0	7853	-+---++	-0.01	0.502	7638	++---+	-0.01	0.648	Carnitine
C10	3805	+???+??	0.01	0.421	3790	+???+?	0.01	0.653	Decanoylcarnitine
C10:1	6573	-+?-++?	0.00	0.933	6551	-+?-++	0.00	0.987	Decenoylcarnitine
C10:2	6575	-+?---?	-0.02	0.066	6553	-+?---	-0.02	0.098	Decadienylcarnitine
C12:1	7853	-++-+--	-0.01	0.300	7638	-++-+-	-0.01	0.365	Dodecenoylcarnitine
C14:1	7852	++--+-+	0.00	0.775	7637	+0--+-	-0.01	0.461	Tetradecenoylcarnitine
C14:1-OH	1453	???-?+?	-0.04	0.121	1453	???-?-	-0.04	0.121	Hydroxytetradecenoylcarnitine
C14:2	6575	-+?-+-?	0.00	0.900	6553	-+?-+-	-0.01	0.657	Tetradecadienylcarnitine
C16:2	805	????-??	-0.01	0.836	805	????-?	-0.01	0.875	Hexadecadienylcarnitine
C18:1	6769	-+?-+-+	-0.01	0.518	6554	-+?-+-	-0.01	0.348	Octadecenoylcarnitine
C18:2	7853	-++--++	0.01	0.626	7638	-++--+	0.00	0.933	Octadecadienylcarnitine
C2	7853	--+---+	-0.03	0.014	7638	--+---	-0.03	0.015	Acetylcarnitine
C3	1448	????++?	0.01	0.780	1448	????++	0.01	0.758	Propionylcarnitine
C3-OH	1449	????--?	-0.10	$2.64x10^{-4}$	1449	????--	-0.10	$2.75x10^{-4}$	Hydroxypropionylcarnitine
C5:1-DC	2120	?+??-??	0.01	0.559	2113	?+??-?	0.01	0.516	Glutaconylcarnitine
C7-DC	6576	-+?---?	-0.02	0.055	6554	-+?---	-0.03	0.017	Pimelylcarnitine
C8	3804	+???+??	0.01	0.459	3789	+???+?	0.01	0.625	Octanoylcarnitine
C8:1	7853	-++--+-	-0.01	0.440	7638	+++--+	0.00	0.916	Octenoylcarnitine
C9	5262	+??-++?	0.04	0.003	5247	+??-++	0.04	0.011	Nonaylcarnitine
Arg	6576	+-?++-?	0.01	0.546	6554	+-?++-	0.01	0.602	Arginine
Gln	7853	+---+++	0.01	0.488	7638	+-+-++	0.00	0.847	Glutamine
Gly	7851	++-+-++	0.01	0.261	7636	+0-+-+	0.00	0.828	Glycine
His	7853	+--+-++	0.01	0.562	7638	+--+-+	0.00	0.834	Histidine
Met	7852	------+	-0.04	$9.20x10^{-5}$	7637	------	-0.05	$7.51x10^{-5}$	Methionine
Orn	7853	+-+--++	-0.01	0.611	7638	+-+--+	-0.01	0.527	Ornithine
Phe	7853	--+-+-+	-0.01	0.363	7638	--+-+-	-0.01	0.597	Phenylalanine
Pro	7852	------+	-0.02	0.183	7637	------	-0.01	0.230	Proline
Ser	7853	++++-++	0.02	0.081	7638	-+++-+	0.01	0.202	Serine
Thr	5963	-+?+?++	0.02	0.241	5748	-+?+?+	0.01	0.340	Threonine

metabolite	Model 1: age + sex				Model 2: age + sex + BMI				metabolite full name
	n	direction*	r	p-value	n	direction*	r	p-value	
Trp	5963	+-?-?++	-0.01	0.619	5748	+-?-?+	-0.01	0.500	Tryptophan
Tyr	7047	----?-+	-0.04	$2.14x10^{-4}$	6832	----?-	-0.04	$8.91x10^{-4}$	Tyrosine
Val	7047	----?++	-0.01	0.541	6829	+---?+	0.00	0.977	Valine
xLeu	7047	----?++	-0.01	0.517	6829	+---?+	0.00	0.952	Leucine / Isoleucine
PC aa C24:0	4006	-??-??+	-0.02	0.290	3798	-??-??	-0.03	0.054	Phosphatidylcholine diacyl C24:0
PC aa C28:1	7853	----+++	-0.01	0.555	7638	----++	-0.01	0.282	Phosphatidylcholine diacyl C28:1
PC aa C30:0	7853	----+++	-0.02	0.070	7638	----++	-0.03	0.021	Phosphatidylcholine diacyl C30:0
PC aa C32:0	7853	----+++	-0.03	0.021	7638	----++	-0.03	0.006	Phosphatidylcholine diacyl C32:0
PC aa C32:1	7851	----+-+	-0.04	$2.44x10^{-4}$	7636	----++	-0.04	$3.38x10^{-4}$	Phosphatidylcholine diacyl C32:1
PC aa C32:2	7853	----+++	-0.01	0.352	7638	----++	-0.02	0.188	Phosphatidylcholine diacyl C32:2
PC aa C32:3	7853	+---+++	0.01	0.495	7638	+---++	0.01	0.623	Phosphatidylcholine diacyl C32:3
PC aa C34:1	7852	----+++	-0.02	0.031	7637	----++	-0.03	0.013	Phosphatidylcholine diacyl C34:1
PC aa C34:2	7853	-----++	-0.02	0.090	7638	-----+	-0.02	0.038	Phosphatidylcholine diacyl C34:2
PC aa C34:3	7853	----+++	-0.02	0.065	7638	----++	-0.02	0.033	Phosphatidylcholine diacyl C34:3
PC aa C34:4	7853	----+++	0.00	0.753	7638	----++	-0.01	0.625	Phosphatidylcholine diacyl C34:4
PC aa C36:0	7853	+---+++	0.01	0.424	7638	----++	0.00	0.977	Phosphatidylcholine diacyl C36:0
PC aa C36:1	7850	----+++	-0.03	0.009	7635	----++	-0.03	0.004	Phosphatidylcholine diacyl C36:1
PC aa C36:2	7853	-----++	-0.02	0.030	7638	-----+	-0.03	0.010	Phosphatidylcholine diacyl C36:2
PC aa C36:3	7852	----+++	-0.02	0.063	7637	----++	-0.02	0.053	Phosphatidylcholine diacyl C36:3
PC aa C36:4	7853	----+++	0.00	0.913	7638	----++	0.00	0.840	Phosphatidylcholine diacyl C36:4
PC aa C36:5	7849	----+++	-0.01	0.508	7634	----++	-0.01	0.229	Phosphatidylcholine diacyl C36:5
PC aa C36:6	7851	+---+++	0.00	0.715	7636	----++	0.00	0.823	Phosphatidylcholine diacyl C36:6
PC aa C38:0	7853	+-+-+++	0.01	0.355	7638	--+-++	0.00	0.735	Phosphatidylcholine diacyl C38:0
PC aa C38:1	836	?????++	0.08	0.026	643	?????+	0.05	0.173	Phosphatidylcholine diacyl C38:1
PC aa C38:3	7852	----+++	-0.03	0.007	7637	----++	-0.02	0.035	Phosphatidylcholine diacyl C38:3
PC aa C38:4	7851	----+++	-0.01	0.514	7636	----++	-0.01	0.646	Phosphatidylcholine diacyl C38:4
PC aa C38:5	7850	+---+++	-0.01	0.652	7635	----++	-0.01	0.342	Phosphatidylcholine diacyl C38:5
PC aa C38:6	7852	+---+-+	0.00	0.987	7637	+---+-	-0.01	0.611	Phosphatidylcholine diacyl C38:6
PC aa C40:1	5770	+0?+?-?	0.01	0.453	5748	++?+?-	0.00	0.740	Phosphatidylcholine diacyl C40:1
PC aa C40:2	7849	-++++++	0.01	0.321	7634	-+++++	0.00	0.758	Phosphatidylcholine diacyl C40:2
PC aa C40:3	7849	-++---+	0.00	0.852	7634	-++---	-0.01	0.435	Phosphatidylcholine diacyl C40:3

metabolite	Model 1: age + sex				Model 2: age + sex + BMI				metabolite full name
	n	direction*	r	p-value	n	direction*	r	p-value	
PC aa C40:4	7850	----+++	-0.01	0.225	7635	----++	-0.01	0.255	Phosphatidylcholine diacyl C40:4
PC aa C40:5	7849	----+++	-0.03	0.022	7634	----++	-0.03	0.017	Phosphatidylcholine diacyl C40:5
PC aa C40:6	7851	----+-+	-0.02	0.073	7636	----+-	-0.02	0.070	Phosphatidylcholine diacyl C40:6
PC aa C42:0	7853	+++++++	0.03	0.020	7638	+++-++	0.02	0.089	Phosphatidylcholine diacyl C42:0
PC aa C42:1	7853	+-+-+++	0.02	0.086	7638	+-+-++	0.01	0.389	Phosphatidylcholine diacyl C42:1
PC aa C42:2	7849	+++++++	0.01	0.236	7634	-0++++	0.00	0.907	Phosphatidylcholine diacyl C42:2
PC aa C42:4	7853	--+-+++	0.00	0.854	7638	--+-++	-0.01	0.599	Phosphatidylcholine diacyl C42:4
PC aa C42:5	7850	--+-+-+	-0.02	0.152	7635	--+-+-	-0.02	0.078	Phosphatidylcholine diacyl C42:5
PC aa C42:6	6764	--?-+-+	0.00	0.999	6549	--?-+-	-0.01	0.478	Phosphatidylcholine diacyl C42:6
PC ae C30:0	6769	+-?-+++	0.01	0.220	6554	--?-++	0.00	0.732	Phosphatidylcholine acyl-alkyl C30:0
PC ae C32:1	7853	----+++	0.00	0.756	7638	----++	-0.01	0.244	Phosphatidylcholine acyl-alkyl C32:1
PC ae C32:2	7853	--+-+++	0.00	0.771	7638	--+-++	-0.01	0.306	Phosphatidylcholine acyl-alkyl C32:2
PC ae C34:0	7853	+---+++	0.00	0.755	7638	----++	-0.01	0.643	Phosphatidylcholine acyl-alkyl C34:0
PC ae C34:1	7852	+---+++	0.00	0.835	7637	----++	-0.01	0.579	Phosphatidylcholine acyl-alkyl C34:1
PC ae C34:2	7853	+---+++	0.01	0.204	7638	----++	0.00	0.808	Phosphatidylcholine acyl-alkyl C34:2
PC ae C34:3	7853	--+++++	0.01	0.538	7638	--+-++	-0.01	0.590	Phosphatidylcholine acyl-alkyl C34:3
PC ae C36:0	7852	+---+++	0.00	0.982	7637	----++	-0.01	0.460	Phosphatidylcholine acyl-alkyl C36:0
PC ae C36:1	7852	+-+-+++	0.03	0.007	7637	+-+-++	0.02	0.058	Phosphatidylcholine acyl-alkyl C36:1
PC ae C36:2	7853	++--+++	0.03	0.014	7638	+---++	0.02	0.132	Phosphatidylcholine acyl-alkyl C36:2
PC ae C36:3	7853	+---+++	0.01	0.301	7638	----++	0.00	0.789	Phosphatidylcholine acyl-alkyl C36:3
PC ae C36:4	7852	+---+++	0.01	0.240	7637	+---++	0.01	0.401	Phosphatidylcholine acyl-alkyl C36:4
PC ae C36:5	7852	----+++	0.00	0.920	7637	----++	-0.01	0.635	Phosphatidylcholine acyl-alkyl C36:5
PC ae C38:0	7851	++--+++	0.01	0.567	7636	++--++	0.00	0.828	Phosphatidylcholine acyl-alkyl C38:0
PC ae C38:1	6854	+-++?-?	0.01	0.250	6829	+-++?-	0.01	0.482	Phosphatidylcholine acyl-alkyl C38:1
PC ae C38:2	7853	+++++++	0.02	0.099	7638	-++-++	0.01	0.402	Phosphatidylcholine acyl-alkyl C38:2
PC ae C38:3	7852	+++-+++	0.03	0.017	7637	+++-++	0.02	0.036	Phosphatidylcholine acyl-alkyl C38:3
PC ae C38:4	7853	+-+-+++	0.04	$9.04x10^{-4}$	7638	+-+-++	0.03	0.005	Phosphatidylcholine acyl-alkyl C38:4
PC ae C38:5	7853	+-+-+++	0.01	0.251	7638	+-+-++	0.01	0.510	Phosphatidylcholine acyl-alkyl C38:5
PC ae C38:6	7852	+---+++	0.01	0.543	7637	+---++	0.00	0.967	Phosphatidylcholine acyl-alkyl C38:6
PC ae C40:1	7852	+-++-++	0.02	0.073	7637	+-++-+	0.01	0.402	Phosphatidylcholine acyl-alkyl C40:1
PC ae C40:2	7853	+-+++++	0.02	0.069	7638	+-++++	0.02	0.147	Phosphatidylcholine acyl-alkyl C40:2

	Model 1: age + sex				Model 2: age + sex + BMI				
**metabolite**	**n**	**direction***	**r**	**p-value**	**n**	**direction***	**r**	**p-value**	**metabolite full name**
PC ae C40:3	7853	+++++++	0.04	0.002	7638	++++++	0.03	0.009	Phosphatidylcholine acyl-alkyl C40:3
PC ae C40:4	7853	+0+++++	0.03	0.004	7638	+-++++	0.03	0.028	Phosphatidylcholine acyl-alkyl C40:4
PC ae C40:5	7853	+-+++++	0.04	0.002	7638	+-++++	0.03	0.019	Phosphatidylcholine acyl-alkyl C40:5
PC ae C40:6	7853	+-+-+++	0.03	0.009	7638	+-+-++	0.02	0.085	Phosphatidylcholine acyl-alkyl C40:6
PC ae C42:0	6768	+0?-+-+	0.00	0.841	6553	-+?-+-	0.00	0.805	Phosphatidylcholine acyl-alkyl C42:0
PC ae C42:1	7852	--+-+++	0.01	0.246	7637	-0+-++	0.01	0.610	Phosphatidylcholine acyl-alkyl C42:1
PC ae C42:2	7852	+-+--++	0.01	0.285	7637	--+--+	0.00	0.906	Phosphatidylcholine acyl-alkyl C42:2
PC ae C42:3	7853	+++++++	0.02	0.052	7638	++++++	0.01	0.414	Phosphatidylcholine acyl-alkyl C42:3
PC ae C42:4	7853	+-+++++	0.02	0.035	7638	+-++++	0.01	0.209	Phosphatidylcholine acyl-alkyl C42:4
PC ae C42:5	6769	+-?++++	0.02	0.084	6554	+-?+++	0.01	0.290	Phosphatidylcholine acyl-alkyl C42:5
PC ae C44:3	7853	--+++++	0.02	0.111	7638	-0++++	0.01	0.518	Phosphatidylcholine acyl-alkyl C44:3
PC ae C44:4	7852	+-+--++	0.01	0.418	7637	+-+---	0.00	0.994	Phosphatidylcholine acyl-alkyl C44:4
PC ae C44:5	7853	+-+--++	0.01	0.212	7638	+-+--+	0.01	0.527	Phosphatidylcholine acyl-alkyl C44:5
PC ae C44:6	7852	++++-++	0.02	0.099	7637	+++--+	0.01	0.353	Phosphatidylcholine acyl-alkyl C44:6
lysoPC a C16:0	7853	++-++++	0.02	0.120	7638	++-+++	0.01	0.417	lysoPhosphatidylcholine acyl C16:0
lysoPC a C16:1	7851	-+--+-+	-0.01	0.368	7636	-+--+-	-0.01	0.207	lysoPhosphatidylcholine acyl C16:1
lysoPC a C17:0	7853	++-++++	0.05	$7.10x10^{-6}$	7638	++-+++	0.04	$4.72x10^{-4}$	lysoPhosphatidylcholine acyl C17:0
lysoPC a C18:0	7853	++--+++	0.02	0.096	7638	+0--++	0.01	0.332	lysoPhosphatidylcholine acyl C18:0
lysoPC a C18:1	7853	++---++	0.01	0.264	7638	-0---+	0.00	0.771	lysoPhosphatidylcholine acyl C18:1
lysoPC a C18:2	7851	++++-++	0.02	0.154	7636	-+++-+	0.00	0.872	lysoPhosphatidylcholine acyl C18:2
lysoPC a C20:3	7853	-+---++	0.00	0.956	7638	-+---+	-0.01	0.620	lysoPhosphatidylcholine acyl C20:3
lysoPC a C20:4	7853	++++-++	0.02	0.065	7638	++++-+	0.01	0.314	lysoPhosphatidylcholine acyl C20:4
lysoPC a C26:0	1314	?+?????	0.02	0.565	1307	?+????	0.02	0.457	lysoPhosphatidylcholine acyl C26:0
lysoPC a C28:0	6766	-0?-+-+	-0.01	0.447	6551	-+?-+-	-0.02	0.175	lysoPhosphatidylcholine acyl C28:0
lysoPC a C28:1	7852	+++-+++	0.02	0.096	7637	-++-++	0.01	0.279	lysoPhosphatidylcholine acyl C28:1
SM (OH) C14:1	7853	+---+++	0.02	0.079	7638	+---++	0.01	0.331	Hydroxysphingomyeline C14:1
SM (OH) C16:1	7047	+-+-?++	0.03	0.011	6832	+-+-?+	0.02	0.053	Hydroxysphingomyeline C16:1
SM (OH) C22:1	7047	+0++?++	0.02	0.129	6829	+-++?+	0.01	0.310	Hydroxysphingomyeline C22:1
SM (OH) C22:2	7047	+-+-?++	0.02	0.051	6829	+-+-?+	0.02	0.213	Hydroxysphingomyeline C22:2
SM (OH) C24:1	7047	+0+-?++	0.01	0.209	6829	+-+-?+	0.01	0.457	Hydroxysphingomyeline C24:1
SM C16:0	7853	--+-+++	0.00	0.864	7638	--+-++	-0.01	0.330	Sphingomyeline C16:0

	Model 1: age + sex				Model 2: age + sex + BMI				
**metabolite**	**n**	**direction***	**r**	**p-value**	**n**	**direction***	**r**	**p-value**	**metabolite full name**
SM C16:1	7853	--+-+++	-0.02	0.066	7638	--+-++	-0.02	0.074	Sphingomyeline C16:1
SM C18:0	7853	--+-+++	0.00	0.933	7638	+-+-++	0.00	0.864	Sphingomyeline C18:0
SM C18:1	7853	--+-+++	0.00	0.822	7638	+-+-++	0.01	0.598	Sphingomyeline C18:1
SM C20:2	7853	+-+-+++	0.03	0.002	7638	+-+-++	0.03	0.003	Sphingomyeline C20:2
SM C24:0	7852	--+++++	0.00	0.906	7637	--+-++	-0.01	0.657	Sphingomyeline C24:0
SM C24:1	7853	--+--++	-0.01	0.402	7638	--+--+	-0.01	0.236	Sphingomyeline C24:1
SM C26:0	5454	+??-+++	0.03	0.043	5246	+??-++	0.02	0.229	Sphingomyeline C26:0
SM C26:1	6539	+?+++++	0.01	0.241	6328	-?+-++	0.01	0.604	Sphingomyeline C26:1
H1	7851	----+--	-0.02	0.073	7636	----++	-0.01	0.363	Hexose

*Order of cohorts in direction column: KORA, NTR, EGCUT, TwinsUK, ERF, LLS, QIMR; Direction of effect represented by - (negative correlation) + (positive correlation) , ? (not included), or not available (0) for each study.

Supplementary Table 3: Study-specific results for age- and sex-adjusted model

	KORA (N=3003)		NTR (N=1314)		EGCUT (N=1084)		TwinsUK (N=810)		ERF (N=806)		LLS (N=643)		QIMR (N=193)	
**metabolite**	**r**	**p-value**	**r**	**p-value**	**r**	**p-value**	**r**	**p-value**	**r**	**p-value**	**r**	**p-value**	**r**	**p-value**
lysoPC a C17:0	0.08	$8.19x10^{-6}$	0.07	0.016	-0.02	0.455	0.01	0.873	0.02	0.597	0.08	0.032	0.09	0.234
Met	-0.05	0.006	-0.04	0.157	-0.01	0.708	-0.01	0.887	-0.13	$1.26x10^{-4}$	-0.04	0.300	0.04	0.537
Tyr	-0.05	0.005	-0.06	0.023	-0.02	0.606	-0.05	0.188	NA	NA	-0.04	0.286	0.05	0.521
PC aa C32:1	-0.06	0.001	-0.05	0.087	-0.04	0.163	-0.06	0.069	0.00	0.925	0.00	0.989	0.05	0.494
C3-OH	NA	NA	NA	NA	NA	NA	NA	NA	-0.15	$3.11x10^{-5}$	-0.03	0.420	NA	NA
PC ae C38:4	0.05	0.003	-0.01	0.723	0.00	0.920	-0.01	0.746	0.06	0.112	0.13	0.001	0.11	0.141
PC ae C40:3	0.04	0.041	0.00	0.918	0.03	0.280	0.01	0.875	0.07	0.048	0.08	0.049	0.09	0.193
PC ae C40:5	0.05	0.009	-0.01	0.798	0.01	0.648	0.02	0.641	0.05	0.170	0.09	0.025	0.09	0.227
SM C20:2	0.02	0.190	-0.04	0.128	0.07	0.029	-0.02	0.667	0.21	$1.04x10^{-9}$	0.00	0.937	0.09	0.224
C9	0.06	0.003	NA	NA	NA	NA	-0.03	0.374	0.03	0.346	0.08	0.052	NA	NA
PC ae C40:4	0.04	0.021	0.00	1.000	0.01	0.654	0.01	0.834	0.03	0.452	0.11	0.006	0.09	0.237
PC aa C38:3	-0.04	0.024	-0.05	0.071	-0.02	0.493	-0.09	0.013	0.02	0.518	0.03	0.483	0.04	0.553
PC ae C36:1	0.03	0.071	-0.01	0.798	0.00	0.894	-0.01	0.718	0.06	0.077	0.12	0.003	0.15	0.035
PC aa C36:1	-0.05	0.013	-0.07	0.016	-0.03	0.283	-0.06	0.092	0.03	0.359	0.05	0.199	0.09	0.210
PC ae C40:6	0.05	0.005	-0.01	0.819	0.01	0.832	-0.01	0.781	0.05	0.176	0.04	0.281	0.10	0.147
SM (OH) C16:1	0.05	0.010	-0.02	0.507	0.02	0.571	-0.01	0.861	NA	NA	0.09	0.021	0.12	0.102
C2	-0.03	0.143	-0.06	0.020	0.03	0.378	-0.08	0.018	-0.01	0.747	-0.02	0.593	0.04	0.603
PC ae C36:2	0.03	0.062	0.00	0.913	-0.01	0.750	-0.01	0.792	0.06	0.104	0.09	0.017	0.12	0.088
PC ae C38:3	0.03	0.105	0.00	0.892	0.01	0.702	-0.03	0.477	0.06	0.092	0.09	0.020	0.10	0.165
PC aa C42:0	0.03	0.155	0.01	0.692	0.06	0.037	0.00	0.968	0.02	0.662	0.04	0.364	0.05	0.515
PC aa C32:0	-0.05	0.005	-0.06	0.036	-0.03	0.360	-0.03	0.451	0.04	0.222	0.05	0.234	0.07	0.324
PC aa C40:5	-0.03	0.074	-0.06	0.039	-0.03	0.396	-0.05	0.137	0.02	0.505	0.01	0.836	0.08	0.262
PC aa C38:1	NA	NA	NA	NA	NA	NA	NA	NA	NA	NA	0.06	0.134	0.14	0.057
PC aa C36:2	-0.04	0.051	-0.07	0.014	-0.02	0.563	-0.03	0.344	-0.01	0.854	0.05	0.170	0.10	0.154
PC aa C34:1	-0.03	0.066	-0.05	0.056	-0.05	0.131	-0.04	0.205	0.00	0.954	0.07	0.091	0.11	0.140
PC ae C42:4	0.04	0.049	-0.01	0.654	0.04	0.169	0.01	0.821	0.00	0.895	0.04	0.292	0.07	0.366
SM C26:0	0.03	0.075	NA	NA	NA	NA	-0.03	0.424	0.05	0.170	0.01	0.744	0.14	0.048
SM (OH) C22:2	0.03	0.144	-0.01	0.786	0.01	0.754	0.00	0.946	NA	NA	0.09	0.016	0.13	0.078
PC ae C42:3	0.02	0.201	0.01	0.699	0.03	0.337	0.01	0.727	0.01	0.837	0.03	0.505	0.12	0.085

	KORA (N=3003)		NTR (N=1314)		EGCUT (N=1084)		TwinsUK (N=810)		ERF (N=806)		LLS (N=643)		QIMR (N=193)	
**metabolite**	**r**	**p-value**	**r**	**p-value**	**r**	**p-value**	**r**	**p-value**	**r**	**p-value**	**r**	**p-value**	**r**	**p-value**
C7-DC	-0.02	0.368	0.03	0.216	NA	NA	-0.09	0.008	-0.06	0.074	-0.04	0.331	NA	NA
PC aa C36:3	-0.04	0.050	-0.04	0.152	-0.03	0.300	-0.05	0.127	0.00	0.890	0.08	0.048	0.09	0.193
lysoPC a C20:4	0.02	0.180	0.02	0.552	0.01	0.834	0.01	0.747	-0.03	0.469	0.07	0.066	0.14	0.055
PC aa C34:3	-0.03	0.076	-0.03	0.223	-0.03	0.274	-0.03	0.361	0.02	0.623	0.02	0.578	0.06	0.392
SM C16:1	-0.04	0.015	-0.07	0.013	0.01	0.765	-0.04	0.249	0.03	0.375	0.07	0.088	0.08	0.250
C10:2	-0.03	0.115	0.04	0.127	NA	NA	-0.09	0.007	-0.01	0.872	-0.06	0.144	NA	NA
PC ae C40:2	0.03	0.091	-0.01	0.772	0.01	0.699	0.00	0.945	0.02	0.655	0.06	0.148	0.08	0.281
PC aa C30:0	-0.04	0.018	-0.06	0.034	-0.04	0.177	-0.01	0.716	0.06	0.075	0.06	0.158	0.08	0.288
H1	-0.02	0.173	-0.03	0.263	-0.01	0.654	-0.04	0.275	0.02	0.555	0.00	1.000	-0.08	0.287
PC aa C40:6	-0.02	0.308	-0.04	0.109	-0.03	0.343	-0.05	0.179	0.05	0.164	-0.05	0.248	0.08	0.294
PC ae C40:1	0.02	0.279	0.00	0.904	0.02	0.601	0.02	0.639	-0.01	0.850	0.07	0.067	0.17	0.020
SM (OH) C14:1	0.02	0.180	-0.03	0.278	-0.01	0.783	0.00	0.972	0.05	0.168	0.09	0.020	0.17	0.017
Ser	0.01	0.711	0.04	0.198	0.00	0.918	0.02	0.555	-0.02	0.563	0.10	0.013	0.11	0.129
PC ae C42:5	0.04	0.047	-0.02	0.398	NA	NA	0.01	0.739	0.02	0.616	0.05	0.186	0.03	0.629
PC aa C42:1	0.02	0.245	0.00	0.909	0.03	0.278	-0.01	0.786	0.02	0.520	0.04	0.288	0.10	0.162
PC aa C34:2	-0.03	0.170	-0.06	0.044	-0.02	0.563	-0.02	0.577	-0.05	0.123	0.09	0.027	0.11	0.132
lysoPC a C18:0	0.03	0.063	0.01	0.856	-0.01	0.821	0.00	0.975	0.01	0.727	0.04	0.262	0.05	0.513
lysoPC a C28:1	0.00	0.840	0.00	0.918	0.02	0.556	-0.03	0.454	0.09	0.007	0.03	0.428	0.20	0.006
PC ae C44:6	0.02	0.186	0.01	0.779	0.04	0.192	0.00	0.888	-0.01	0.812	0.03	0.517	0.04	0.620
PC ae C38:2	0.01	0.736	0.01	0.819	0.01	0.740	0.00	0.990	0.04	0.222	0.08	0.045	0.12	0.105
PC ae C44:3	-0.01	0.566	0.00	0.924	0.03	0.309	0.01	0.800	0.08	0.030	0.06	0.132	0.19	0.010
lysoPC a C16:0	0.02	0.296	0.03	0.319	-0.01	0.662	0.00	0.890	0.02	0.666	0.05	0.223	0.06	0.389
C14:1-OH	NA	NA	NA	NA	NA	NA	-0.07	0.034	NA	NA	0.00	0.964	NA	NA
SM (OH) C22:1	0.01	0.613	NA	1.000	0.01	0.629	0.00	0.890	NA	NA	0.10	0.015	0.10	0.185
PC aa C42:5	-0.03	0.085	-0.01	0.692	0.01	0.787	-0.03	0.434	0.03	0.373	-0.06	0.101	0.06	0.401
lysoPC a C18:2	0.01	0.476	0.02	0.507	0.01	0.839	0.02	0.533	-0.04	0.229	0.07	0.067	0.14	0.057
Pro	-0.01	0.614	-0.03	0.321	-0.02	0.523	-0.03	0.344	0.00	0.952	-0.03	0.428	0.08	0.269
PC ae C34:2	0.01	0.551	-0.02	0.477	-0.01	0.690	-0.01	0.728	0.04	0.291	0.11	0.004	0.14	0.061
SM (OH) C24:1	0.01	0.620	NA	1.000	0.02	0.412	0.00	0.966	NA	NA	0.06	0.107	0.06	0.396
PC ae C44:5	0.02	0.233	-0.02	0.504	0.05	0.115	-0.02	0.562	-0.02	0.631	0.06	0.113	0.04	0.596
PC ae C30:0	0.01	0.616	-0.01	0.609	NA	NA	-0.02	0.663	0.05	0.137	0.06	0.119	0.12	0.101

	KORA (N=3003)		NTR (N=1314)		EGCUT (N=1084)		TwinsUK (N=810)		ERF (N=806)		LLS (N=643)		QIMR (N=193)	
**metabolite**	**r**	**p-value**	**r**	**p-value**	**r**	**p-value**	**r**	**p-value**	**r**	**p-value**	**r**	**p-value**	**r**	**p-value**
PC aa C40:4	-0.04	0.036	-0.03	0.346	0.00	0.901	-0.05	0.156	0.06	0.087	0.04	0.352	0.08	0.296
PC aa C42:2	0.00	0.838	0.00	0.913	0.02	0.581	0.02	0.590	0.00	0.922	0.04	0.344	0.15	0.032
PC ae C36:4	0.01	0.643	-0.03	0.219	-0.01	0.744	-0.01	0.753	0.05	0.194	0.13	0.001	0.13	0.066
Thr	0.00	0.935	0.01	0.819	NA	NA	0.02	0.598	NA	NA	0.09	0.018	0.06	0.391
SM C26:1	0.00	0.858	NA	NA	0.02	0.588	0.00	0.981	0.05	0.199	0.01	0.720	0.11	0.131
PC ae C42:1	-0.01	0.614	0.00	0.904	0.04	0.194	0.00	0.902	0.05	0.174	0.04	0.356	0.17	0.015
PC ae C38:1	0.03	0.122	-0.01	0.764	0.02	0.485	0.02	0.510	NA	NA	-0.03	0.422	NA	NA
PC ae C38:5	0.02	0.313	-0.05	0.098	0.00	0.914	-0.01	0.711	0.04	0.278	0.10	0.014	0.11	0.143
Gly	0.03	0.132	0.01	0.654	-0.02	0.607	0.01	0.770	-0.01	0.807	0.02	0.642	0.02	0.742
lysoPC a C18:1	0.02	0.400	0.02	0.559	-0.01	0.727	-0.01	0.776	-0.02	0.516	0.07	0.060	0.11	0.112
PC ae C42:2	0.00	0.793	0.00	0.896	0.03	0.316	0.00	0.989	-0.01	0.825	0.05	0.205	0.14	0.055
PC aa C24:0	-0.03	0.072	NA	NA	NA	NA	-0.02	0.562	NA	NA	NA	NA	0.25	0.001
C12:1	-0.01	0.616	0.03	0.339	0.00	0.985	-0.09	0.011	0.00	0.900	-0.01	0.797	-0.13	0.082
PC ae C36:3	0.01	0.553	-0.02	0.400	-0.01	0.786	-0.03	0.447	0.05	0.183	0.10	0.012	0.10	0.185
PC aa C40:2	0.00	0.994	0.00	0.909	0.04	0.239	0.00	0.893	0.01	0.843	0.02	0.697	0.14	0.058
PC aa C32:2	-0.02	0.208	-0.02	0.502	-0.04	0.243	-0.03	0.343	0.06	0.091	0.01	0.710	0.10	0.171
PC aa C38:0	0.01	0.742	-0.01	0.707	0.02	0.608	-0.02	0.625	0.04	0.236	0.03	0.397	0.10	0.159
Phe	-0.01	0.498	-0.04	0.168	0.02	0.593	-0.04	0.243	0.02	0.630	0.00	0.959	0.05	0.477
lysoPC a C16:1	-0.01	0.519	0.01	0.749	-0.02	0.603	-0.05	0.163	0.01	0.853	-0.02	0.601	0.05	0.521
SM C24:1	-0.03	0.146	-0.02	0.425	0.01	0.651	-0.01	0.788	0.00	0.987	0.03	0.399	0.03	0.650
PC ae C44:4	0.02	0.360	-0.01	0.610	0.05	0.075	-0.01	0.686	-0.03	0.428	0.01	0.758	0.04	0.581
C10	0.01	0.506	NA	NA	NA	NA	-0.07	0.037	0.02	0.639	NA	NA	NA	NA
PC aa C36:0	0.00	0.843	-0.01	0.717	0.00	0.994	-0.02	0.643	0.04	0.265	0.03	0.431	0.18	0.011
C8:1	-0.01	0.757	0.02	0.562	0.01	0.848	-0.06	0.106	-0.02	0.519	0.01	0.877	-0.10	0.183
lysoPC a C28:0	-0.02	0.382	NA	1.000	NA	NA	-0.04	0.296	0.04	0.244	-0.08	0.034	0.19	0.009
PC aa C40:1	0.02	0.370	NA	1.000	NA	NA	0.01	0.733	NA	NA	0.00	0.945	NA	NA
C8	0.01	0.498	NA	NA	NA	NA	-0.09	0.014	0.01	0.762	NA	NA	NA	NA
Gln	0.04	0.029	-0.09	0.002	0.00	0.918	-0.02	0.481	0.02	0.597	0.08	0.034	0.05	0.521
PC aa C32:3	0.00	0.934	-0.04	0.150	-0.01	0.802	-0.03	0.466	0.10	0.007	0.07	0.092	0.09	0.209
C0	0.00	0.938	0.02	0.440	-0.02	0.566	-0.07	0.063	-0.03	0.368	0.01	0.876	0.05	0.464
PC aa C36:5	0.00	0.844	-0.02	0.517	-0.06	0.063	0.00	0.888	0.01	0.785	0.00	0.984	0.17	0.018

	KORA (N=3003)		NTR (N=1314)		EGCUT (N=1084)		TwinsUK (N=810)		ERF (N=806)		LLS (N=643)		QIMR (N=193)	
**metabolite**	**r**	**p-value**	**r**	**p-value**	**r**	**p-value**	**r**	**p-value**	**r**	**p-value**	**r**	**p-value**	**r**	**p-value**
PC aa C38:4	-0.01	0.498	-0.05	0.083	-0.01	0.706	-0.04	0.228	0.04	0.267	0.06	0.145	0.11	0.141
xLeu	-0.01	0.626	-0.03	0.269	0.00	0.947	-0.02	0.612	NA	NA	0.03	0.399	0.04	0.581
C18:1	-0.01	0.532	0.00	0.892	NA	NA	-0.07	0.063	0.00	0.937	-0.01	0.798	0.17	0.016
PC ae C34:3	-0.01	0.424	-0.02	0.559	0.00	0.887	0.01	0.863	0.05	0.157	0.08	0.047	0.10	0.156
Val	-0.01	0.515	-0.03	0.250	-0.01	0.823	-0.01	0.702	NA	NA	0.05	0.242	0.08	0.284
PC ae C38:6	0.01	0.619	-0.04	0.169	-0.01	0.664	-0.02	0.614	0.05	0.129	0.05	0.175	0.14	0.045
Arg	0.02	0.301	-0.04	0.180	NA	NA	0.01	0.825	0.05	0.187	0.00	0.913	NA	NA
PC aa C28:1	-0.01	0.651	-0.08	0.004	-0.02	0.461	-0.01	0.719	0.04	0.208	0.07	0.088	0.17	0.021
C5:1-DC	NA	NA	0.02	0.373	NA	NA	-0.04	0.234	-0.01	0.849	NA	NA	NA	NA
His	0.03	0.159	-0.04	0.106	0.00	0.892	0.05	0.155	-0.06	0.114	0.04	0.362	0.10	0.181
lysoPC a C26:0	NA	NA	0.02	0.565	NA	NA	NA	NA	NA	NA	NA	NA	NA	NA
PC ae C38:0	0.01	0.546	0.01	0.806	-0.03	0.407	-0.01	0.709	0.02	0.639	0.01	0.823	0.14	0.045
Orn	0.01	0.676	-0.06	0.037	0.01	0.706	-0.03	0.411	-0.03	0.336	0.04	0.316	0.11	0.141
Trp	0.00	0.913	-0.05	0.084	NA	NA	0.00	0.902	NA	NA	0.02	0.604	0.05	0.534
C18:2	0.00	0.987	0.00	0.929	0.04	0.176	-0.03	0.360	-0.02	0.545	0.01	0.842	0.18	0.013
PC aa C38:5	0.00	0.920	-0.04	0.142	-0.04	0.201	-0.03	0.405	0.02	0.494	0.04	0.304	0.15	0.041
PC aa C36:6	0.00	0.841	-0.01	0.764	-0.03	0.295	-0.02	0.519	0.06	0.066	0.00	0.902	0.16	0.030
PC aa C34:4	-0.02	0.385	-0.02	0.522	-0.04	0.185	-0.02	0.515	0.06	0.095	0.05	0.182	0.13	0.082
PC ae C34:0	0.01	0.691	-0.03	0.244	-0.04	0.240	-0.03	0.448	0.05	0.171	0.08	0.041	0.09	0.203
PC ae C32:1	-0.01	0.570	-0.03	0.306	0.00	0.883	-0.02	0.533	0.01	0.824	0.06	0.151	0.11	0.137
PC ae C32:2	-0.02	0.403	-0.04	0.105	0.01	0.854	-0.03	0.357	0.05	0.185	0.05	0.213	0.15	0.031
C14:1	0.01	0.671	0.00	0.935	-0.01	0.858	-0.09	0.014	0.01	0.688	0.00	0.963	0.07	0.302
C3	NA	NA	NA	NA	NA	NA	-0.05	0.129	0.00	0.945	0.01	0.732	NA	NA
SM C18:1	0.00	0.921	-0.04	0.185	0.02	0.413	-0.05	0.126	0.07	0.060	0.04	0.353	0.07	0.341
PC ae C34:1	0.00	0.983	-0.02	0.407	-0.02	0.461	-0.03	0.344	0.03	0.347	0.09	0.025	0.08	0.269
C16:2	NA	NA	NA	NA	NA	NA	NA	NA	-0.01	0.836	NA	NA	NA	NA
PC ae C42:0	0.00	0.908	NA	1.000	NA	NA	-0.02	0.632	0.04	0.287	-0.04	0.302	0.10	0.152
PC aa C40:3	-0.02	0.380	0.00	0.935	0.01	0.679	-0.01	0.840	0.00	0.969	0.00	0.933	0.13	0.082
PC aa C42:4	-0.03	0.166	-0.01	0.631	0.03	0.293	0.00	0.962	0.04	0.306	0.03	0.420	0.14	0.051
SM C16:0	-0.03	0.122	-0.03	0.331	0.02	0.572	-0.01	0.730	0.02	0.479	0.09	0.017	0.08	0.257
C14:2	0.00	0.924	0.03	0.210	NA	NA	-0.07	0.036	0.03	0.474	-0.02	0.662	NA	NA

	KORA (N=3003)		NTR (N=1314)		EGCUT (N=1084)		TwinsUK (N=810)		ERF (N=806)		LLS (N=643)		QIMR (N=193)	
**metabolite**	**r**	**p-value**	**r**	**p-value**	**r**	**p-value**	**r**	**p-value**	**r**	**p-value**	**r**	**p-value**	**r**	**p-value**
SM C24:0	-0.03	0.133	-0.01	0.616	0.01	0.623	0.00	0.986	0.04	0.245	0.05	0.181	0.03	0.637
PC aa C36:4	-0.01	0.705	-0.04	0.119	-0.02	0.426	-0.02	0.615	0.02	0.498	0.10	0.013	0.13	0.062
PC ae C36:5	-0.01	0.508	-0.03	0.213	-0.03	0.341	0.00	0.921	0.04	0.246	0.10	0.014	0.15	0.033
SM C18:0	0.00	0.823	-0.04	0.163	0.01	0.695	-0.03	0.381	0.06	0.105	0.04	0.365	0.07	0.329
C10:1	0.00	0.923	0.03	0.363	NA	NA	-0.07	0.049	0.02	0.538	0.03	0.482	NA	NA
lysoPC a C20:3	0.00	0.923	0.02	0.388	-0.01	0.627	-0.04	0.285	-0.04	0.254	0.06	0.129	0.10	0.166
PC ae C36:0	0.01	0.696	-0.01	0.600	-0.04	0.159	-0.01	0.722	0.01	0.872	0.03	0.430	0.14	0.046
PC aa C38:6	0.01	0.716	-0.03	0.338	-0.02	0.474	-0.02	0.584	0.04	0.280	0.00	0.906	0.13	0.071
PC aa C42:6	-0.01	0.695	-0.02	0.467	NA	NA	-0.02	0.529	0.08	0.023	-0.03	0.458	0.11	0.139

Supplementary Table 4: Top findings of partial correlation meta-analysis results of LTL and metabolites, including and excluding the TwinsUK cohort, adjusted for age and sex

	Analysis including TwinsUK				Analysis excluding TwinsUK				
**Metabolite**	**N**	**direction***	**r**	**p-value**	**N**	**direction**	**r**	**p-value**	**Metabolite full name**
lysoPC a C17:0	7853	++-**+**+++	0.05	$7.1x10^{-6}$	7043	++-+++	0.06	$2.8x10^{-6}$	Lysophosphatidylcholine acyl C17:0
Met	7852	---**-**--+	-0.04	$9.2x10^{-5}$	7042	-----+	-0.05	$4.5x10^{-5}$	Methionine
Tyr	7047	---**-**?-+	-0.04	$2.1x10^{-4}$	6237	---?-+	-0.04	$5.4x10^{-4}$	Tyrosine
PC aa C32:1	7851	---**-**+-+	-0.04	$2.4x10^{-4}$	7041	---+-+	-0.04	$1.1x10^{-3}$	Phosphatidylcholine diacyl C32:1
C3-OH	1449	???**?**--?	-0.10	$2.6x10^{-4}$	1449	???--?	-0.10	$2.6x10^{-4}$	Hydroxypropionylcarnitine
PC ae C38:4	7853	+-+**-**+++	0.04	$9.0x10^{-4}$	7043	+-++++	0.04	$3.0x10^{-4}$	Phosphatidylcholine acyl-alkyl C38:4

* Order of cohorts in direction column: KORA, NTR, EGCUT, TwinsUK (in red and bold), ERF, LLS, QIMR; Direction of effect represented by - (negative correlation) + (positive correlation) or ? (not included) for each study

Supplementary Table 5: Top findings of partial correlation meta-analysis results of LTL and metabolites, including and excluding the QIMR cohort, adjusted for age and sex

	Analysis including QIMR				Analysis excluding QIMR				
**Metabolite**	**n**	**direction***	**r**	**p-value**	**n**	**direction***	**r**	**p-value**	**Metabolite full name**
lysoPC a C17:0	7853	++-+++**+**	0.05	$7.1x10^{-6}$	7660	++-+++	0.05	$1.3x10^{-5}$	Lysophosphatidylcholine acyl C17:0
Met	7852	------**+**	-0.04	$9.2x10^{-5}$	7659	------	-0.05	$5.0x10^{-5}$	Methionine
Tyr	7047	----?-**+**	-0.04	$2.1x10^{-4}$	6854	----?-	-0.05	$1.1x10^{-4}$	Tyrosine
PC aa C32:1	7851	----+-**+**	-0.04	$2.4x10^{-4}$	7658	----+-	-0.04	$1.3x10^{-4}$	Phosphatidylcholine diacyl C32:1
C3-OH	1449	????--**?**	-0.10	$2.6x10^{-4}$	1449	????--	-0.10	$2.6x10^{-4}$	Hydroxypropionylcarnitine
PC ae C38:4	7853	+-+-++**+**	0.04	$9.0x10^{-4}$	7660	+-+-++	0.04	$1.8x10^{-3}$	Phosphatidylcholine acyl-alkyl C38:4

* Order of cohorts in direction column: KORA, NTR, EGCUT, TwinsUK, ERF, LLS, QIMR (in red and bold); Direction of effect represented by - (negative correlation) + (positive correlation) or ? (not included) for each study
